# Impact of SGLT2 inhibitors on patient outcomes: a network meta-analysis

**DOI:** 10.1186/s12933-023-02035-8

**Published:** 2023-10-27

**Authors:** Jui-Yi Chen, Heng-Chih Pan, Chih-Chung Shiao, Min-Hsiang Chuang, Chun Yin See, Tzu-Hsuan Yeh, Yafei Yang, Wen-Kai Chu, Vin-Cent Wu

**Affiliations:** 1 Division of Nephrology, Department of Internal Medicine, Chi-Mei Medical Center, Tainan, Taiwan; 2 Department of Health and Nutrition, Chia Nan University of Pharmacy and Science, Tainan, Taiwan; 3 Division of Nephrology, Department of Internal Medicine, Keelung Chang Gung Memorial Hospital, Keelung, Taiwan; 4 Chang Gung University College of Medicine, Taoyuan, Taiwan; 5 Graduate Institute of Clinical Medicine, College of Medicine, National Taiwan University, Taipei, Taiwan; 6 Community Medicine Research Center, Keelung Chang Gung Memorial Hospital, Keelung, Taiwan; 7 Division of Nephrology, Department of Internal Medicine, Camillian Saint Mary’s Hospital Luodong; and Saint Mary’s Junior College of Medicine, Nursing and Management, Yilan, Taiwan; 8 Division of Nephrology, Department of Internal Medicine, National Cheng Kung University Hospital, College of Medicine, National Cheng Kung University, Tainan, Taiwan; 9 Division of Nephrology, Department of Internal Medicine, Everan Hospital, Taichung, Taiwan; 10 Department of Internal Medicine, National Taiwan University Hospital, 7 Chung-Shan South Road, Zhong-Zheng District, Taipei, 100 Taiwan; 11 National Taiwan University Hospital Study Group of ARF, NSARF, Taipei, Taiwan; 12 Taiwan Primary Aldosteronism Investigators, TAIPAI, PAC, Taipei, Taiwan

**Keywords:** SGLT2 inhibitor, Diabetes, Heart failure, Chronic kidney disease, Network meta-analysis

## Abstract

**Background:**

A comprehensive network meta-analysis comparing the effects of individual sodium-glucose cotransporter 2 (SGLT2) inhibitors on patients with and without comorbidities including diabetes mellitus (DM), heart failure (HF), and chronic kidney disease (CKD) has not been previously conducted.

**Methods:**

We searched PubMed, Embase, Cochrane, and ClinicalTrials.gov for randomized controlled trials up to March 28, 2023. Network meta-analysis using a random-effects model was conducted to calculate risk ratios (RRs). Risk of Bias tool 2.0 was used to assess bias, and CINeMA to assess the certainty of evidence. In the subgroup analysis, the SGLT2 inhibitors were classified into highly (dapagliflozin, empagliflozin, and ertugliflozin) and less selective SGLT2 inhibitors (canagliflozin and sotagliflozin).

**Results:**

A total of fourteen trials with 75,334 patients were analyzed. Among these, 40,956 had taken SGLT2 inhibitors and 34,378 had not. One of the main results with particular findings was empagliflozin users had a significantly lower risk of all-cause death compared to dapagliflozin users in DM population (RR: 0.81, 95% CI 0.69–0.96). In HF population, sotagliflozin users had a borderline significantly lower risk of CV death or hospitalization for HF (HHF) than dapagliflozin users (RR: 0.90, 95% CI 0.80–1.01). In non-HF population, those who used canagliflozin had a significantly lower risk of CV death or HHF compared with those who used dapagliflozin (RR: 0.75, 95% CI 0.58–0.98). At last, for HF patients, those who used less selective SGLT2 inhibitors had a significantly lower risk of MACEs compared to those who used highly selective SGLT2 inhibitors (RR: 0.75, 95% CI 0.62–0.90).

**Conclusions:**

Our network meta-analysis revealed that empagliflozin users with diabetes experienced a lower risk of dying from any cause than those using dapagliflozin. Additionally, canagliflozin users demonstrated a reduced risk of cardiovascular death or HHF compared to dapagliflozin users in those without HF. In HF patients, less selective SGLT2 inhibitors showed superior CV composite outcomes, even surpassing the performance of highly selective SGLT2 inhibitors.

*Trial registration*: PROSPERO [CRD42022361906].

**Supplementary Information:**

The online version contains supplementary material available at 10.1186/s12933-023-02035-8.

## Background

Sodium-glucose cotransporter 2 (SGLT2) inhibitors were originally developed as antidiabetic agents [1] but were later found to have cardiovascular (CV) and renal protective effects. Clinical trials such as EMPA-REG [2], CANVAS [3], and DECLARE-TIMI 58 [4] demonstrated their benefits in reducing major adverse cardiac events (MACEs) and heart failure hospitalizations(HHF) in type 2 diabetes mellitus (DM) patients. Furthermore, evidence suggests these CV protective effects extend to non-DM patients as highlighted by the DAPA-HF [5] and EMPEROR-Reduced trials [6].

Besides CV protection, SGLT2 inhibitors have been shown to preserve kidney function, as evidenced by trials like CREDENCE [7], DAPA-CKD [8], and EMPA-KIDNEY [9], indicating their benefits in chronic kidney disease (CKD) patients irrespective of DM status.

However, SGLT2 inhibitors have diverse receptor selectivity [10]. For example, dapagliflozin, empagliflozin and ertugliflozin are selective SGLT2 inhibitors, but canagliflozin and sotagliflozin have been shown to have weak selectivity for SGLT2 over SGLT1 [11]. In contrast to SGLT2, SGLT1 receptors are especially expressed in the myocardium [12]. Thus, the specific effects of different classes of SGLT2 inhibitors or even each drug on outcomes are still not clear, and head-to-head comparisons of the currently available SGLT2 inhibitors are lacking.

Numerous RCTs highlight the diverse benefits of SGLT2 inhibitors on patient outcomes across different comorbidities [13–18]. While prior meta-analyses [17, 19] explored the class effects of these drugs, they lacked direct comparisons between individual SGLT2 inhibitors. A 2022 meta-analysis encompassed 13 RCTs [19], yet its insights into the effects on CKD patients, especially non-diabetic ones, were constrained due to data limitations. A recent global RCT revealed empagliflozin's effect on HF patients [20]. In our updated network meta-analysis (NMA), we compiled all pertinent RCT data to systematically review and evaluate individual SGLT2 inhibitors across diverse patient groups, emphasizing their organ-protective roles and offering specific pharmaceutical insights.

## Methods

In alignment with the Preferred Reporting Items for Systematic Reviews and Meta-Analyses (PRISMA) statement [21] and Cochrane methods [22], we conducted a systematic review and NMA of RCTs to assess the impact of SGLT2 inhibitors on the risks of mortality, CV death, CV death or HHF, MACEs, kidney function progression, AKI, and renal-specific composite outcomes for patients with and without DM, CKD, and HF. In addition, we explored side effects like ketoacidosis, lower limb amputation, mycotic genital infection, hypoglycemia, urinary tract infection, and bone fracture. The systematic review protocol was meticulously planned in advance and registered in PROSPERO [CRD42022361906].

### Study search strategy and literature search

We conducted a comprehensive search for RCTs published before March 28, 2023 in PubMed, Embase, Cochrane, and ClinicalTrials.gov, and only retrieved double-blind trials so that placebo groups could be included in the NMA. No limitations geographic location, or publication year were imposed in the search strategy. The language was restricted on publishing in English. The search was guided by terms such as “sodium-glucose cotransporter 2 inhibitors”, “SGLT2 inhibitors”, “dapagliflozin”, “empagliflozin”, “canagliflozin”, “ertugliflozin”, “sotagliflozin”, “mortality”, “death” and “cardiovascular death”. To enhance the robustness of our search and minimize potential biases, two investigators (JY Chen and TH Yeh) independently searched the published RCTs. We then obtained the full text of the selected papers for quality assessment and data synthesis. When required, we contacted the papers’ authors to acquire more information.

### Inclusion and exclusion criteria

We considered RCTs to be eligible if they met the following criteria: (a) population: adult patients (≥ 18 years old) with/without DM, HF, and CKD separately; (b) intervention: patients administrated SGLT2 inhibitors; (c) control group as patients who did not take SGLT2 inhibitors; (d) outcome: risks of all-cause mortality, CV death, CV death or HHF, kidney function progression, and AKI. The exclusion criteria were studies: (1) with patients < 18 years old; (2) lacking information regarding SGLT2 inhibitors or outcomes; and (3) other than RCTs, including observational studies, reviews, narrative reviews, letters, editorials, conference abstracts, case reports, etc. Two investigators (JY Chen and VC Wu) scrutinized the titles and abstracts of the preliminary set of articles and then delved deeper into the selected studies for the final analysis. All disagreements were resolved through discussion with a third investigator (CC Shiao).

### Data extraction and outcomes

For each eligible study, we extracted general information (first author, year of publication, year of study, study title, study design, sample size), baseline demographic and clinical characteristics of the participants (types of SGLT2 inhibitors, comorbidities), intervention/exposure (SGLT2 inhibitors/placebo treatment), and outcome data including the number of cases and events for each group. Two trials (CANVAS and SCORED) lacked specific event numbers, so we derived them from the incidence rate, which is the number of patients per certain unit of person-years. Outcomes were all-cause death, CV death, CV death or HHF, MACEs, kidney function progression, AKI, renal-specific composite outcomes and diabetic complications (including ketoacidosis, lower limbs amputation, urinary tract infection, mycotic genital infection, hypoglycemia, and bone fracture). 3-points MACEs encompassed CV death, ischemic stroke or myocardial infarction. The renal composite outcome was defined as a composite of kidney progression and AKI.

### Risk of bias and quality assessment

We employed the Risk of Bias tool 2.0 to assess the possible biases of the enrolled studies. We used the CINeMA (Confidence in Network Meta-Analysis) web application to assess the certainty of evidence, which allows for confidence in the results to be graded as high, moderate, low, and very low. The per-protocol evaluation was chosen in the intervention adherence section owing to matching the methods of our included studies. Any discrepancies in data extraction and quality evaluation were settled through collaborative discussions among authors.

### Subgroup analysis

We hypothesized that the SGLT2 selectivity of SGLT2 inhibitors may play a role on patient outcomes observed among different studies. We divided the five SGLT2 inhibitors into two groups based on their relative SGLT2 selectivity: highly selective SGLT2 inhibitors (dapagliflozin, empagliflozin, and ertugliflozin) and less selective SGLT2 inhibitors (canagliflozin and sotagliflozin) [10, 11].

### Data synthesis and statistical analysis

Initially, a set of network analysis was conducted using R (version 4.2.2, R Project for Statistical Computing) software, and frequentist statistics was used for the meta-analysis to approach a random-effect NMA. In addition, a network map was used to elucidate associations between the SGLT2 inhibitor and placebo groups with MetaInsight (V4.0.0) [23]. The number of patients in each group was proportionally expressed by the size of nodes, and the width of the edges was proportional to the number of studies that compared individuals who did and did not receive SGLT2 inhibitors. Surface under the cumulative ranking (SUCRA) was reported for treatment effects. The SUCRA value is the ratio of the area under the cumulative ranking curve to the entire area in the plot, which represents that a treatment is successful without uncertainty [24]. No evaluation of inconsistency between the direct and indirect effects was required since no direct comparisons between any two SGLT2 inhibitors in the included trials.

**Fig. 1:**
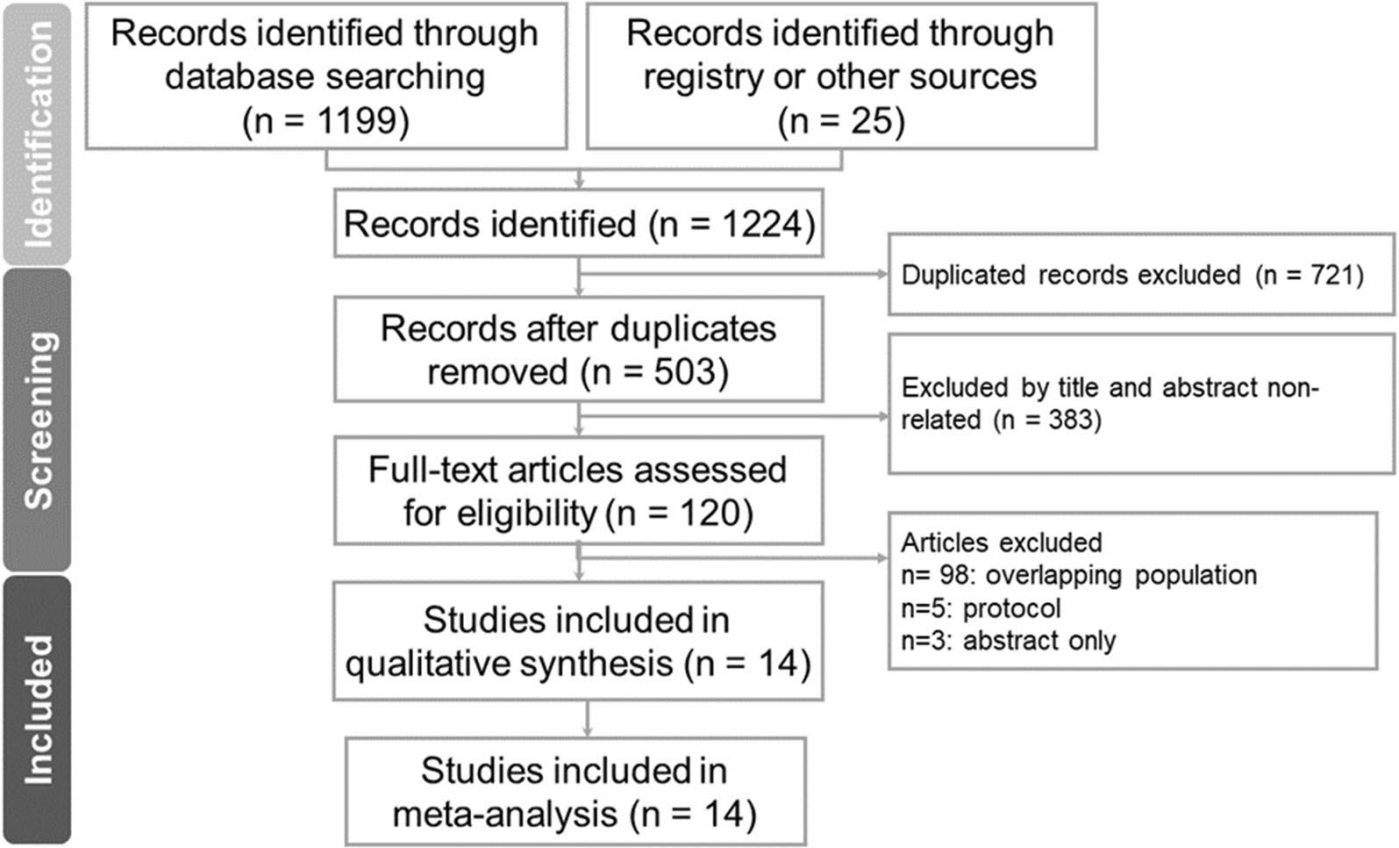
PRISMA diagram of studies included in the meta-analysis. *PRISMA* preferred reporting items for systematic reviews and meta-analyses

In addition, we included studies incrementally into the analysis according to the publication year to conduct trial sequential analysis (TSA) for a path of estimates for each pairwise comparison, and also to provide a methodological framework for updating the NMA [25]. The sequential monitoring boundaries (Copenhagen Trial Unit, Centre for Clinical Intervention Research, Denmark, software 0·9·5·10 Beta software) were also used to control type I and type II errors. The conventional non-superiority boundaries were set at significance levels of 0.05 and a power of 90%. The α-spending boundaries were calculated using the O'Brien-Fleming procedure. TSA tested the mortality, CV death, CV death or HHF, kidney function progression, and AKI in the DM/non-DM patients, CV death or HHF in the CKD/non-CKD patients, and CV death or HHF, and MACEs in the HF/non-HF patients to assess the temporal and cumulative effect of each article on the results of the present study. We checked publication bias using funnel plots along with the Egger’s test [26]. At last, we rated the certainty of evidence according to Cochrane methods and the GRADE approach (Fig. 1).

## Results

### Study search outcomes and included patients

From an extensive search, we identified 1,224 studies. Of these, 721 were duplicates and 503 were removed based on the eligibility. A further 383 studies were excluded due to irrelevant titles and abstracts. An additional 98 articles had overlapping populations, five were solely protocols and three were only abstracts were also excluded. Ultimately, 14 studies including 75,334 patients with complete data of interested outcomes were selected for the final meta‐analysis (Additional file 1: Figure S1).

The characteristics of the included 14 studies, which were published from 2017 to 2022, are illustrated in Table 1. Among the 14 studies, four enrolled patients with empagliflozin [6, 9, 20, 27], and another three included patients with dapagliflozin [8, 28, 29]. In addition, two studies included patients with HF with reduced ejection fraction (HFrEF) [6, 28], and another two studies included patients with HF with preserved ejection fraction (HFpEF) [27, 29]. In addition, three studies included patients with CKD. Among the included patients, 40,956 had taken SGLT2 inhibitors (SGLT2 inhibitor group) and 34,378 had not (placebo group) (Table 1).

**Table 1:** Summary of the baseline characteristics of the included studies

Study (year)	SGLT2i	Population	Patients (SGLT2i/Placebo) (n)	DM/non-DM (SGLT2i, Placebo) (n)	HF/non-HF (SGLT2i, Placebo) (n)	CKD/non-CKD (SGLT2i, Placebo) (n)
EMPEROR-REDUCED [6] (2020)	Empagliflozin 10 mg once daily	HFrEF	1863/1867	(927, 926)/NR	(1863, 1867)/(0, 0)	(969, 960)/(893, 906)
EMPA-REG [2] (2015)	Empagliflozin 10 mg or 25 mg once daily	Type 2 diabetes	4687/2333	(4687, 2333)/NR	NR/NR	(1196, 605)/(3449, 1718)
EMPEROR-PRESERVED [27] (2021)	Empagliflozin 10 mg once daily	HFpEF	2997/2991	(1465, 1471)/NR	(2997, 2991)/(0, 0)	(1504, 1484)/(1493, 1505)
EMPLUSE [20] (2021)	Empagliflozin 10 mg once daily	acute de novo or decompensated chronic HF	265/265	NR/NR	(265, 265)/(0, 0)	NR/NR
EMPA-KIDNEY [9] (2022)	Empagliflozin 10 mg once daily	CKD	3304/3305	(1525, 1515)/(1779, 1790)	NR/NR	(3304, 3305)/(0, 0)
DECLARE-TIMI 58 [4] (2009)	Dapagliflozin 10 mg once daily	Type 2 diabetes	8582/8578	(8582, 8578)/(0, 0)	(852, 872)/(7730, 7706)	(606, 659)/(7975, 7919)
DAPA-HF [5] (2020)	Dapagliflozin 10 mg once daily	HFrEF	2373/2371	(1075, 1064)/(1298, 1307)	(2373, 2371)/(0, 0)	(962, 964)/(1410, 1406)
DAPA-CKD [8] (2020)	Dapagliflozin 10 mg once daily	CKD	2152/2152	(1455, 1451)/(697, 701)	NR/NR	(2152, 2152)/(0, 0)
DELIVER [29] (2022)	Dapagliflozin 10 mg once daily	HFrEF or HFpEF	3131/3132	(1578, 1572)/(1551, 1558)	(3131, 3132)/(0, 0)	(1516, 1554)/(1615, 1577)
CANVAS [3] (2017)	Canagliflozin 300 mg or 100 mg once daily	Type 2 diabetes	5795/4347	(5795, 4347)/(0, 0)	(803, 658)/(4992, 3689)	NR/NR
CREDENCE [7] (2019)	Canagliflozin 100 mg once daily	Type 2 diabetes	2202/2199	(2202, 2199)/(0, 0)	(329, 323)/(1873, 1876)	(1297, 1295)/(905, 904)
SOLOIST-WHF [49] (2021)	Sotagliflozin 200 mg once daily	Type 2 diabetes	608/614	(608, 614)/(0, 0)	(608, 614)	NR/NR
SCORED [48] (2021)	Sotagliflozin 200 mg once daily	Type 2 diabetes + CKD	5292/5292	(5292, 5292)/(0,0)	(1640, 1643)/(3652, 3649)	(5292, 5292)/(0,0)
VERTIS CV [59] (2020)	Ertugliflozin 5 mg or 15 mg once daily	Type 2 diabetes	5499/2747	(5499, 2747)/(0,0)	(1288, 671)/(4207, 2074)	(1288, 671)/(4207, 2074)

*CKD* chronic kidney disease, *HF* heart failure, *HFpEF* heart failure with preserved ejection fraction, *HFrEF* heart failure with reduced ejection fraction, *NR* not reported, *SGLT2i* sodium-glucose cotransporter 2 inhibitor

### Risk of bias assessment

We found low risks of bias in randomization, deviations from the intended interventions, attrition, outcome measurements, and selective reporting domains in all of the 14 included trials. Detailed results of the risk-of-bias assessments are provided in the supplementary materials (Additional file 1: Figure S2).

### Main results of this meta-analytic study

#### Death in the DM subgroup

**Fig. 2:**
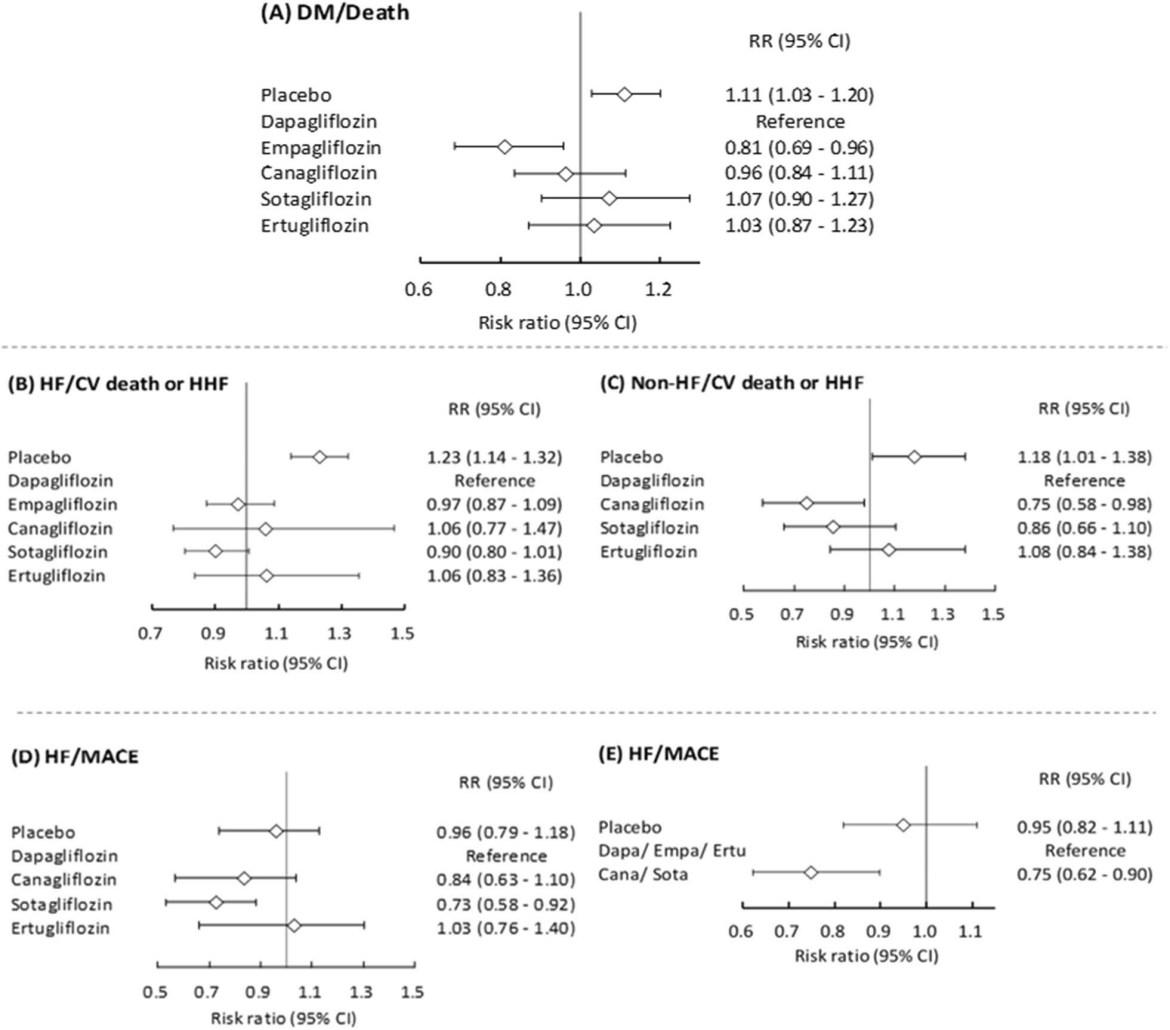
Forest plot showing **A** empagliflozin users had significantly lower risk of death compared to dapagliflozin users among DM population **B** Sotagliflozin users had a lower risk of cardiovascular death and HHF in HF patients **C** Canagliflozin users had significantly lower risk of cardiovascular death and HHF in non-HF patients **D** Sotagliflozin users had a significant lower risk of MACE than placebo users, but other SGLT2 inhibitors didn’t for HF population **E** Less selective SGLT2 inhibitors(canagliflozin and sotagliflozin) users had significant lower risk of MACE compared with highly selective SGLT-2 inhibitors(dapagliflozin, empagliflozin, and ertugliflozin) users for HF patients. *AKI* acute kidney injury, *DM* Diabetes Mellitus, *HF* Heart failure, *HHF* hospitalization heart failure, *MACE* Major adverse cardiac events, *SGLT2* Sodium–Glucose Cotransporter 2

**Fig. 3:**
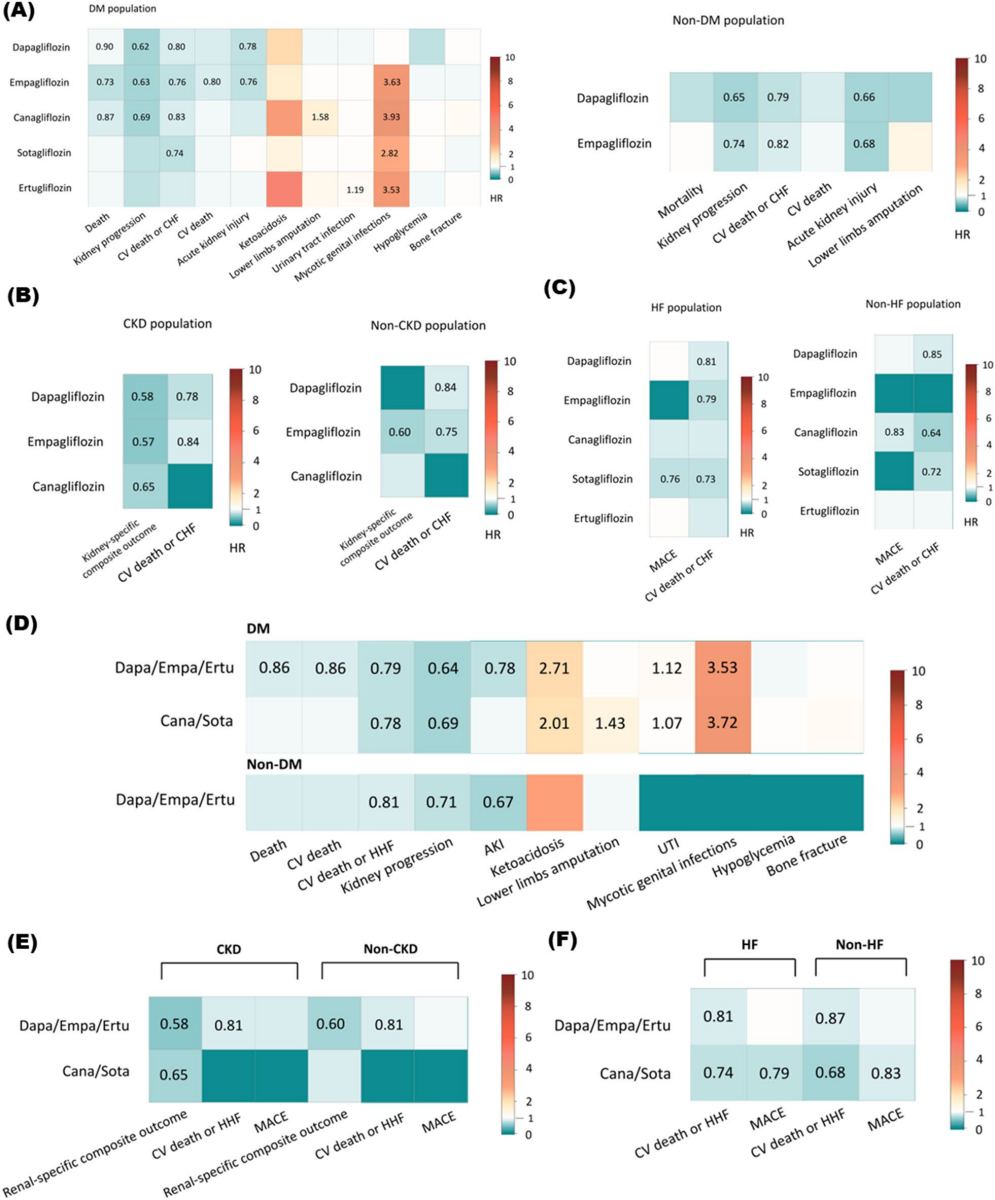
Heatmap plot depicting pairwise comparisons (row vs. column) of risk ratio between the types of SGLT2 inhibitors and outcomes. **A** Dapagliflozin, Empagliflozin, Canagliflozin, Sotagliflozin, Ertugliflozin for the risk of death, kidney function progression, cardiovascular death or HHF, cardiovascular death, AKI, ketoacidosis, lower limbs amputation, UTI, Mycotic genital infections, Hypoglycemia, Bone fracture among DM patients. Dapagliflozin, Empagliflozin for the risk death, kidney function progression, cardiovascular death or HHF, cardiovascular death, AKI, lower limbs amputation among non-DM patients **B** Dapagliflozin, Empagliflozin, Canagliflozin for the risk of kidney specific composite outcomes, cardiovascular death or HHF among CKD patients. Dapagliflozin, Empagliflozin, Canagliflozin for the risk of kidney specific composite outcomes, cardiovascular death or HHF among non-CKD patients. **C** Dapagliflozin, Empagliflozin, Canagliflozin, Sotagliflozin, Ertugliflozin for the risk of MACE, cardiovascular death or HHF among HF patients. Dapagliflozin, Empagliflozin, Canagliflozin, Sotagliflozin, Ertugliflozin for the risk of MACE, cardiovascular death or HHF among non-HF patients. **D** Highly selective SGLT-2 inhibitors (dapagliflozin, empagliflozin, and ertugliflozin) and less selective SGLT-2 inhibitors (canagliflozin and sotagliflozin) for the risk of death, cardiovascular death, cardiovascular death or HHF, kidney function progression, AKI, Ketoacidosis, lower limbs amputation, UTI, mycotic genital infection, hypoglycemia, bone fracture for DM and non-DM patients. **E** Highly selective SGLT-2 inhibitors (dapagliflozin, empagliflozin, and ertugliflozin) and less selective SGLT-2 inhibitors (canagliflozin and sotagliflozin) for the risk of renal specific composite outcomes, cardiovascular death or HHF, and MACE for CKD and non-CKD patients. **F** Highly selective SGLT-2 inhibitors (dapagliflozin, empagliflozin, and ertugliflozin) and less selective SGLT-2 inhibitors (canagliflozin and sotagliflozin) for the risk of cardiovascular death or HHF, and MACE for HF and non-HF patients. The contents of the diagonal are the values of the risk ratio. Red depicts a higher risk ratio, and Green depicts a lower risk ratio. Colored blocks without value means p value more than 0.05 while there is no available data in white blocks. *AKI* acute kidney injury, *Cana* canagliflozin, *CKD* Chronic kidney disease, *Dapa* dapagliflozin, *DM* Diabetes Mellitus, *Empa* empagliflozin, *Ertu* ertugliflozin, *HF* Heart failure, *HHF* Hospitalization for heath failure, *MACE* Major adverse cardiac events, *SGLT2* Sodium–Glucose Cotransporter 2, *Sota* sotagliflozin, *UTI* Urinary tract infection

A total of 73,123 patients with DM were retrieved from 11 studies, of whom 5,724 died during follow-up (mortality rate 8.0%). Empagliflozin users had a significantly lower risk of death compared to dapagliflozin users (RR: 0.81, 95% CI 0.69–0.96) (Figs. 2A, 3A, Additional file 1: Figure S1A). SUCRA showed that empagliflozin was associated with a lower risk of death than dapagliflozin (Additional file 1: Figure S2A). TSA showed that the cumulative z curve reached the benefit boundary without attaining required information size (Additional file 1: Figure S3A).

#### CV death or HHF in the HF subgroup

**Fig. 4:**
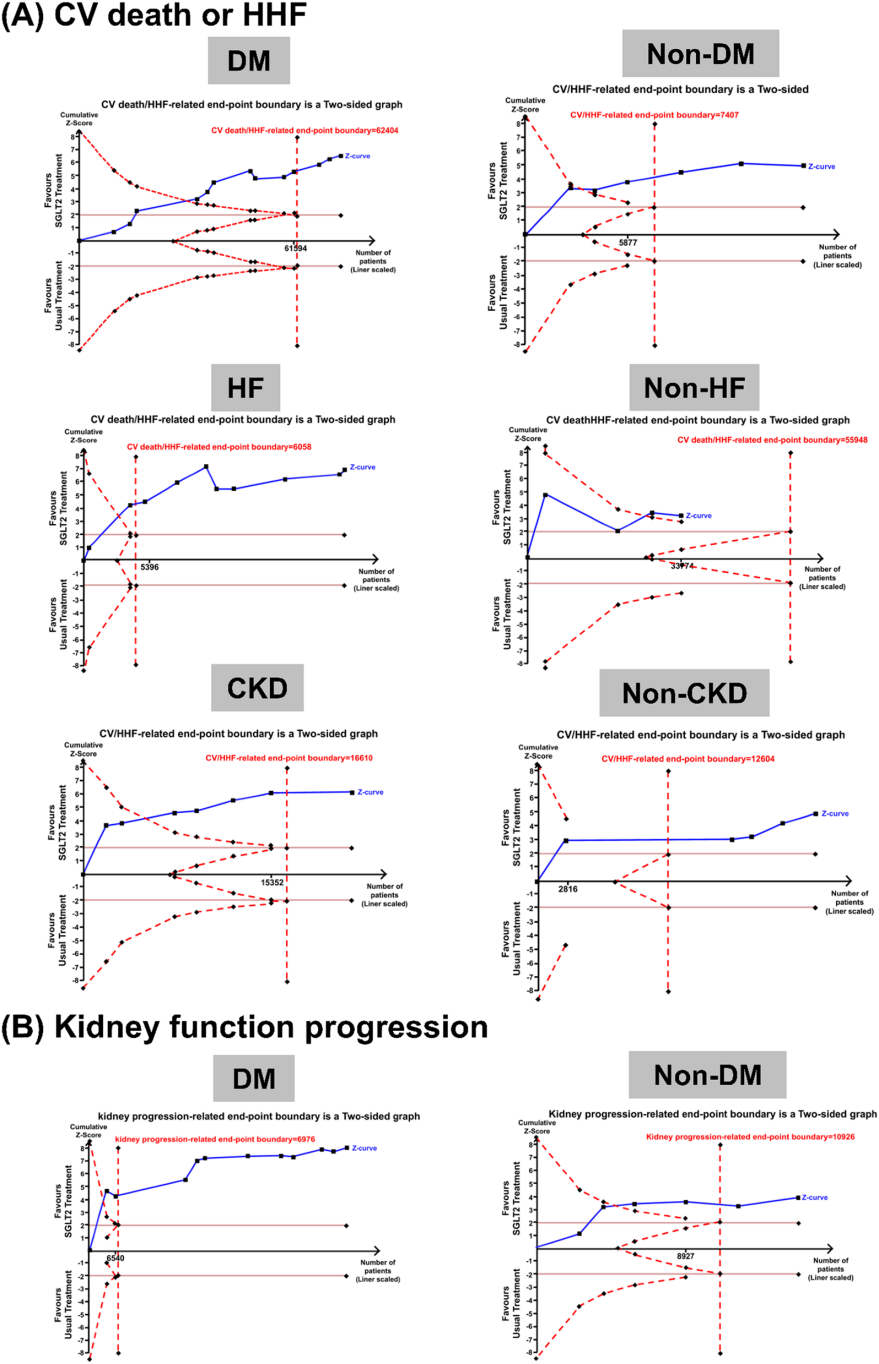
Sequential network meta-analyses over **A** Cardiovascular death or hospitalization for heart failure among DM/non-DM, HF/non-HF, CKD/non-CKD patients **B** Kidney function progression, AKI for DM/non-DM patients with SGLT2 inhibitors versus placebo. *AKI* acute kidney injury, *CKD* Chronic kidney disease, *DM* Diabetes Mellitus, *HF* Heart failure, *SGLT2* Sodium–Glucose Cotransporter 2

A total of 30,094 patients with HF were retrieved from 10 studies, of whom 2,759 (9.2%) developed the composite outcome of CV death or HHF during follow-up. Sotagliflozin users (RR: 0.73, 95% CI 0.67–0.80), dapagliflozin users (RR: 0.81, 95% CI 0.76–0.88), and empagliflozin (RR: 0.79, 95% CI 0.73–0.86) users had significantly lower risks of CV death or HHF than placebo users (Fig. 3C). Sotagliflozin users had a borderline significantly lower risk of CV death or HHF than dapagliflozin users with borderline significance (RR: 0.90, 95% CI 0.80–1.01) (Fig. 2B). SUCRA ranking demonstrated that sotagliflozin was associated with a lower risk of CV death or HHF (Additional file 1: Figure S2D). The cumulative z curve exceeded the boundary of benefit and required information size (Fig. 4A).

#### CV death or HHF in the non-HF subgroup

A total of 32,774 patients without HF were retrieved from four studies, of whom 1,721 (5.3%) developed the composite outcome of CV death or HHF during follow-up. Dapagliflozin (RR: 0.85, 95% CI 0.72–0.99), canagliflozin (RR: 0.64, 95% CI 0.51–0.79), and sotagliflozin (RR: 0.72, 95% CI 0.59–0.89) were associated with lower risks of CV death or HHF (Fig. 3C). Among the non-HF patients, those who used canagliflozin had a significantly lower risk of CV death or HHF compared with those who used dapagliflozin (RR: 0.75, 95% CI 0.58–0.98) (Fig. 2C). SUCRA ranking revealed that canagliflozin was associated with a lower risk of CV death or HHF (Additional file 1: Figure S2E). The cumulative z curve by TSA exceeded the boundary of benefit without sufficient sample size (Fig. 4A).

#### MACEs in the HF and non-HF subgroups

A total of 7,018 patients with MACEs were retrieved from five studies, of whom 1,522 (21.7%) had MACEs during follow-up. The incidence rate of MACE was 21.7%. Among HF patients, sotagliflozin users had a significant lower risk of MACEs than placebo users (RR: 0.76, 95% CI 0.67–0.85), but dapagliflozin (RR: 1.04, 95% CI: 0.85–1.27), canagliflozin (RR: 0.87, 95% CI 0.72–1.05) and ertugliflozin users (RR: 1.07, 95% CI 0.85–1.34) did not (Fig. 3C). Sotagliflozin users also had a significantly lower risk of MACEs than dapagliflozin users (RR: 0.73, 95% CI 0.58–0.92) (Fig. 2D). SUCRA ranking revealed that sotagliflozin was associated with a lower risk of MACEs (Additional file 1: Figure S2F). Among non-HF patients, only canagliflozin users had significant lower risk of MACE compare with placebo (RR: 0.83, 95% CI 0.71–0.95) (Fig. 3C).

#### Highly vs. less selective SGLT2 inhibitors

The SGLT2 inhibitors were classified into highly (dapagliflozin, empagliflozin, and ertugliflozin) and less selective SGLT2 inhibitors (canagliflozin and sotagliflozin). The detailed results of each outcome in the DM and non-DM groups were provided in the supplements (Additional file 1: Figure S6). For HF patients, those who used less selective SGLT2 inhibitors had a significantly lower risk of MACEs compared to those who used highly selective SGLT2 inhibitors (RR: 0.75, 95% CI 0.62–0.90) (Fig. 2E and Additional file 1: Figure S2G, S7D). The clinical outcomes of DM/non-DM, CKD/non-CKD, HF/non-HF groups who received highly selective SGLT2 inhibitors versus less selective SGLT2 inhibitors are demonstrated in Fig. 3D, E and F.

### Other results

The complete results of all possible comparisons are detailed and provided in the supplements (Additional file 1: Figures S1–S8). The clinical outcomes of the patients with/without DM, HF and CKD who received the SGLT2 inhibitors empagliflozin, dapagliflozin, canagliflozin, sotagliflozin and ertugliflozin are listed in Table 2. By using the data of diabetes and non-diabetes populations, the funnel plot and Egger’s test did not detect any apparent publication bias (Additional file 1). At last, the confidence in the evidence according to CINeMA is demonstrated in Additional file 1.

**Table 2:** Qualitative summary of the network meta-analysis

Population	Outcome	Empagliflozin	Dapagliflozin	Canagliflozin	Sotagliflozin	Ertugliflozin	TSA
DM	Death	Lowest risk	Lower risk	Lower risk	Without significance	Without significance	Non-conclusion
	CV death	Without significance	Lowest risk	Without significance	Without significance	Without significance	Non-conclusion
	CV death or HHF	Lower risk	Lower risk	Lower risk	Lower risk	Without significance	Conclusion*
	Kidney function progression	Lower risk	Lowest risk	Lower risk	Without significance	Without significance	Conclusion*
	AKI	Lower risk	Lower risk	Lower risk without significance	Without significance	Without significance	Conclusion*
	Ketoacidosis	Without significance	Without significance	Higher risk without significance	Without significance	Higher risk without significance	
	Lower limb amputation	Without significance	Without significance	Highest risk	Without significance	Higher risk without significance	
	UTI	Without significance	Without significance	Higher risk without significance	Without significance	Highest risk	
	Mycotic genital infections	Higher risk	Highest risk	Higher risk	Higher risk	Higher risk	
	Hypoglycemia	Without significance	Lower risk without significance	Without significance	Without significance	Without significance	
	Bone fracture	Without significance	Without significance	Higher risk without significance	Without significance	Without significance	
Non-DM	Death	Higher risk than Dapagliflozin without significant difference	Lower risk	NR	NR	NR	Non-conclusion
	CV death	Lower risk	Lower risk	NR	NR	NR	Non-conclusion
	CV death or HHF	Lower risk	Lower risk	NR	NR	NR	Conclusion*
	Kidney function progression	Lower risk	Lower risk	NR	NR	NR	Conclusion*
	AKI	Lower risk	Lower risk	NR	NR	NR	Conclusion*
	Lower limbs amputation	Without significance	Without significance	NR	NR	NR	
HF	MACEs		Without significance	Lower risk without significance	Lowest risk	Without significance	Non-conclusion
	CV death or HHF	Lower risk	Lower risk	Without significance	Lowest risk	Without significance	Conclusion*
Non-HF	MACEs		Lower risk without significance	Lowest risk		Without significance	Non-conclusion
	CV death or HHF	Lower risk	Lower risk	Lowest risk		Without significance	Non-conclusion
CKD	Kidney-specific composite outcome	Lowest risk	Lower risk	Lower risk			
	CV death or HHF	Lower risk	Lowest risk				Conclusion*
	Renal-specific composite outcome	Lowest risk		Lower risk without significance			
Non-CKD	Kidney- specific composite outcome	Lowest risk					
	CV death or HHF	Lowest risk		Lower risk			Conclusion*

*AKI* acute kidney injury, *CKD* chronic kidney disease, *CV* cardiovascular, *HF* heart failure, *HHF* hospitalization for heart failure, *MACEs* major adverse cardiac events, *TSA* trial sequential analysis, *UTI* urinary tract infection. *Conclusion: TSA showed that the cumulative z curve reached the benefit boundary with attaining required information size

## Discussion

To the best of our knowledge, this is the most up-to-date systematic review and NMA to comprehensively investigate the impact of individual SGLT2 inhibitors on the clinical outcomes among patients with and without DM, CKD, and HF. In our systematic review of 14 RCT studies including 75,334 patients, the overall incidence rates of the CV composite outcome, MACEs and all-cause mortality were 5.7%, 5.3%, and 5.5%, respectively. The pooled results of the included RCTs indicated that dapagliflozin and empagliflozin were associated with significantly lower risks of the CV composite outcome overall, and lower risks of kidney outcomes in the patients with CKD, DM, and those without DM, compared to a placebo. Among patients with DM, both canagliflozin and sotagliflozin were linked to diminished risks of CV events. Specifically, canagliflozin correlated with a reduced risk of kidney-related outcomes but, conversely, an elevated risk of lower limb amputation when compared to a placebo. In addition, dapagliflozin, empagliflozin, and canagliflozin users had notably lower risks of mortality compared to placebo users, and the beneficial effect of empagliflozin was significantly better than dapagliflozin. In the CKD patients, canagliflozin was associated with a lower risk of kidney outcomes, and in the HF patients, sotagliflozin users was associated with the lowest risk of the CV composite outcome. In addition, within the HF patients, less selective SGLT2 inhibitors were associated with a significantly lower risk of MACEs compared with highly selective SGLT2 inhibitors.

### The rationale of renoprotective and cardioprotective effects of SGLT2 inhibitors

The human kidney filters up to 162 g of glucose daily and reabsorbs 80–90% of this filtered glucose from the urine through the SGLT2 located in the proximal tubules. The kidney-protective effect of SGLT2 inhibitors may be due to their ability to inhibit glucose and sodium reabsorption, which subsequently results in a reduction in intra-glomerular pressure [30, 31]. SGLT2 inhibitors not only inhibit cardiac sodium transporters and alter ion homeostasis but also reduce inflammation and oxidative stress [32]. Additionally, they attenuate cardiac microvascular ischemia/reperfusion injury through improving endothelial function and mitochondrial homeostasis [33]. This off-target effect contributes to cardiovascular benefit [32], a reduction in frailty among patients with diabetes and hypertension [34, 35]. The natriuretic, glycosuric and osmotic diuretic effects of SGLT2 inhibitors have been shown to reduce cardiac preload, lung and systemic congestion, resulting in a cardioprotective effect [31]. Moreover, our findings reveal that in individuals suffering from type 2 diabetes and experiencing acute myocardial infarction, the utilization of SGLT2 inhibitors yielded favorable outcomes, manifesting in reduced inflammatory burden and diminished infarct sizes when compared to alternative oral anti-diabetic agents, irrespective of glucose control [36].

In response to SGLT2 inhibition, the expression or activity of SGLT1 might be elevated in renal proximal tubules to compensate and avoid hypernatriuresis and diuresis^32^. Several previous studies have demonstrated the renoprotective and cardioprotective effects of SGLT2 inhibitors in various clinical settings [13–18].

### Comparisons of different SGLT2 inhibitors in the patients with CKD, DM, and those without DM

SGLT2 inhibitors have been demonstrated to reduce the risk of composite outcomes, including myocardial infarction, stroke, and all-cause mortality, when compared to dipeptidyl peptidase-4 inhibitors [37]. Furthermore, it is noteworthy that SGLT-2 inhibitors demonstrated a higher efficacy in reducing HHF when compared to GLP-1 receptor agonists in patients with type 2 diabetes [38]. In the current meta-analysis, TSA demonstrated that SGLT2 inhibitors had conclusive renoprotective effects in the patients with and without DM, and cardioprotective effects in the patients with and without DM as well as those with CKD. Our results also showcased that dapagliflozin and empagliflozin, the two SGLT2 inhibitors with the highest SGLT2 selectivity, were associated with superior CV death or HHF and renal outcomes in these patients than placebo, which is in agreement with previous meta-analyses [14, 15, 17]. Moreover, in the patients with DM, the beneficial effect of empagliflozin on mortality was significantly greater than that of dapagliflozin. SGLT2 inhibitors also have gained significant market attention for treating type 2 diabetes, with dapagliflozin being a notable example. However, a study revealed that despite expectations, dapagliflozin inhibited glucose reabsorption by only 30–50% [39]. In contrast, empagliflozin, which exhibited higher concentration levels in the market following administration, might explain its observed lower risk of DM death in our research compared to dapagliflozin [39]. These findings suggest that SGLT2 inhibitors with greater selectivity for SGLT2 may be advantageous for these patients.

### Comparisons of different SGLT2 inhibitors in the patients with HF

Our results also demonstrated the cardioprotective effects of SGLT2 inhibitors in the patients with HF in TSA analysis. We found that sotagliflozin, dapagliflozin, and empagliflozin were all associated with a reduction in the risk of CV death and HHF, however only sotagliflozin was found to reduce the risk of MACEs when compared to placebo. This greater cardioprotective effect of sotagliflozin may be attributed to its lower selectivity for SGLT2 and greater inhibition of SGLT-1, as SGLT1 is widely expressed in the heart and is considered to be the major isoform of cardiac SGLTs [40–42]. Our findings are in line with the evidence from a recent meta-analysis conducted by Tager et al., who demonstrated an inverse relationship between the benefits on cardiovascular outcomes and SGLT2 selectivity in patients with HF [10]. In patients with a recent coronary event and drug naïve dysglycemia, empagliflozin has been shown to enhance insulin sensitivity [43]. This is supported further by the observation that empagliflozin modifies specific microRNA patterns in frail HFpEF patients with diabetes, indicating improved endothelial function [44]. Moreover, treatment with SGLT2i significantly optimizes cardiac volumes, elevates LV systolic function, and reduces LV mass [45]. Specifically, following a recent myocardial infarction, empagliflozin treatment resulted in a notable reduction in NT-proBNP and brought about significant enhancements in echocardiographic functional and structural parameters [46]. Collectively, these findings advocate for the safe and efficacious use of empagliflozin as a primary glucose-lowering treatment in high cardiovascular risk patients with newly diagnosed dysglycemia. Treatment with SGLT2i significantly reversed cardiac volumes, improving LV systolic function and LV mass.

### Comparisons of highly selective SGLT2 inhibitors and less selective SGLT2 inhibitors

In the literature, the use of less selective SGLT2 inhibitors has been shown to reduce the upregulation of SGLT1, which further contributes to greater reductions in sodium and glucose reuptake [47], and could result in reductions in cardiac preload, afterload and a better cardioprotective effect [48, 49]. Through double labeling of the human heart with aquaporin 1, the human SGLT1(hSGLT1) protein has been localized in heart capillaries. The discovery of hSGLT1 in these new locations suggests that this transporter has several functions beyond the kidneys. These functions include fluid absorption in the lungs, providing energy to Clara cells, regulating the secretion of enteroendocrine cells, and releasing glucose from heart capillaries [50]. Notably, during ischemia–reperfusion injury (IRI), the expression of SGLT1 at the plasma membrane remains consistent regardless of dietary conditions, while SGLT2 is not detected in the hearts [42]. This evidence points towards the potential superior cardiac prognosis associated with less selective SGLT2 inhibitors that also target SGLT1. In the present study, we found that the less selective SGLT2 inhibitors were linked to a notably reduced risk of MACEs and exhibited more promising cardioprotective effects than highly selective SGLT2 inhibitors in the patients with HF. Conversely, in other population, both highly and less selective SGLT2 inhibitors had beneficial effects on CV and kidney composite outcomes. However, only the highly selective SGLT2 inhibitors were correlated with a decreased risk of AKI in the patients with DM. These observations underscore the significance of varying SGLT2 selectivity on both CV and renal outcomes. Nevertheless, additional comprehensive studies are imperative to clarify the optimal application of SGLT2 inhibitors in patients with diverse comorbidities.

### Side effects of SGLT2 inhibitors

SGLT2 inhibitors are generally perceived as safe, however, our findings indicate that ertugliflozin increased the risk of UTI in the patients with DM. Additionally, we observed that all SGLT2 inhibitors, with the exception of dapagliflozin were associated with increased risks of mycotic genital infections, and that canagliflozin was associated with an increased risk of lower limb amputation in in the patients with DM. A preceding NMA identified a positive relationship between rapid drug action and potent efficacy of SGLT2 inhibitors in reducing HbA1c in the patients with DM [51]. Considering that ertugliflozin is the most potent rapidly absorbed drug and that dapagliflozin is excreted most slowly by the kidneys among the three SGLT2 inhibitors, our findings further demonstrate a positive relationship between rapid drug action and higher risk of adverse events with SGLT2 inhibitors in patients with DM [51, 52].

Our findings highlighted that the canagliflozin users had a high risk of limb amputation. Canagliflozin exhibits less selective for SGLT2 than other SGLT2 inhibitors, leading to a more pronounced inhibitory effect on SGLT1. Lin et al. reported that the risk of lower limb amputation was slightly increased in canagliflozin users [53]. This result is corroborated by other studies demonstrating reduced intravascular volume and hypotension due to insufficient compensatory effects of SGLT1 [48, 49]. Consequently, consistent monitoring of lower limb perfusion for SGLT2 inhibitor users with peripheral artery occlusive disease or those on diuretics could help in mitigating the onset of lower limb complications [54].

### Strengths and limitations

In the present study we analyzed all outcomes from previous RCTs, especially a recently published RCT which has never been integrated in previous meta-analyses.

We used standard Cochrane protocols in comparison with the previous reports and used the GRADE approach to rate the certainty of evidence [55]. We analyzed six different SGLT2 inhibitors and populations separately, and demonstrated the importance of SGLT2 selectivity from different SGLT2 inhibitors with regards to the beneficial effects in different populations. We presented the results from NMA followed by those from sequential TSA, in which dynamic updates of the effect size helped to corroborate the NMA results and estimate the uncertainty of evidence by depicting the trend and making allowance for multiple tests. NMA can provide evidence on comparative effectiveness, which is valuable for clinical decision-making because it allows for comparisons of SGLT2 inhibitors that have rarely been directly compared in recent head-to-head trials. Despite the encouraging results observed in this study, several potential limitations should be recognized. First, the data extracted for the subgroup analyses from some enrolled studies lacked comprehensive information regarding baseline characteristics with verification bias. The risks of CV and renal outcomes differed in the placebo groups in the enrolled RCTs, and a greater reduction in adverse events may have been seen in the patients at a higher risk and further biased our conclusions [14]. Second, the definitions of CKD and HF varied between the studies, which could introduce imprecision the pooled effect estimates. For example, patients with HF are classified into HFrEF, HFmrEF (heart failure with mid-range EF), and HFpEF. HFmrEF is milder than HFrEF with a lower cardiovascular risk. Evidence from post hoc and subgroup analyses of randomized clinical trials, as well as a trial involving an SGLT2 inhibitor, suggests that drugs effective for HFrEF might also benefit HFmrEF patients [56]. However, the effects of SGLT2i may vary between HFrEF and HFpEF. Nonetheless, our conclusions were drawn from studies with different study designs and different clinical situations. Further research is certainly necessary to improve precision medicine. Third, for outcomes reported for all SGLT2 inhibitor users or placebo groups, we could not extract data for subgroup analysis, e.g. DM/non-DM, HF/non-HF, CKD/non-CKD. Ascertainment bias may exist because of the different follow-up periods in the included studies. When studying long-term outcomes, death may act as a competing risk. This means that if a patient dies from causes unrelated to the primary outcome of interest, they can no longer experience the event we are studying. As a result, their death can potentially bias our estimates if not properly accounted for. Fourth, some included RCTs were terminated early due to loss of funding or interim analysis [7, 8, 48, 49], and therefore healthy survivor bias is possible. To maintain statistical power, the primary endpoints of some RCTs were changed but the power to show a difference between the trial groups was not recalculated, which may have biased the results toward the benefit of the trial drugs and further biased the findings of the current study [48, 49]. Fifth, we also found trends of benefits on CV and kidney outcomes with ertugliflozin, but they were not statistically significant. Taking into account the similar mechanisms and SGLT2 selectivity of ertugliflozin to dapagliflozin and empagliflozin, the relatively weak beneficial effects of ertugliflozin may be due to the fact that only one of the 14 included RCTs examined the effect of ertugliflozin. Sixth, most of the included RCTs examined the additive effects of SGLT2 inhibitors in patients receiving renin-angiotensin system blockers, which may also have biased the results toward the benefit of the candidate drugs and further biased our study findings^4^. Seventh, doses of the SGLT2 inhibitors were not fixed in the enrolled studies and we combined trials with different doses, which may have confounded our conclusions comparing the efficacy of different SGLT2 inhibitors [48, 49, 57, 58]. Eighth, we artificially grouped the SGLT2 inhibitors into weak and high SGLT2 selectivity, which may have introduced indication bias. Ninth, some analyses were not conclusive because of the limited number of RCTs enrolled. For example, our results showed that both sotagliflozin and canagliflozin were associated with better CV outcomes than placebo in the non-HF patients, whereas canagliflozin was associated with an even lower risk of CV death or HHF than dapagliflozin in these patients. However, TSA suggested that more studies are needed to make final conclusions about these RCTs evaluating the effect of SGLT2 inhibitors on CV composite outcomes in non-HF patients. Tenth,, in real-world scenarios, no patient receives a placebo treatment. Consequently, deriving clinical evidence from such conditions may not be reflective of actual clinical practice. I contend that this represents a significant limitation of such studies.

## Conclusions

Dapagliflozin and empagliflozin were associated with significantly lower risks of kidney events and the CV composite outcome than placebo in all populations. Canagliflozin was associated with significantly lower risks of kidney progression and CV composite outcome, but a higher risk of limb amputation compared with placebo in the patients with DM. In the HF patients, less selective SGLT2 inhibitors were associated with the best CV composite outcomes, even outperforming highly selective SGLT2 inhibitors.

### Supplementary Information

**Additional file 1: Figure S1.** Network geometry and forest plot of selected results, including (A)DM patients with death; (B)non-DM patients with cardiovascular death or HHF; (C)Non-DM patients with AKI; (D)CKD patients with cardiovascular death or HHF and (E)non-CKD patients with cardiovascular death or HHF. **Figure S2.** Areas under the cumulative ranking curves for of selected results, including individual SGLT2 inhibitors with regard to (A) death, (B) AKI among DM patients, (C) AKI among non-DM patients, (D) cardiovascular death or HHF among HF patients, (E) cardiovascular death or HHF among non-HF patients, (F) MACE among HF patients (G) MACE among HF patients for highly selective SGLT2 inhibitors (dapagliflozin, empagliflozin, ertugliflozin) and less selective SGLT2 inhibitors (canagliflozin, sotagliflozin). **Figure S3.** Sequential network meta-analyses of selected results, including (A)Mortality among DM/non-DM, (B) AKI for DM/non-DM, (C) MACE for HF/non-HF patients with SGLT2 inhibitors versus placebo. **Figure S4.** The complete results of forest plots and SUCRA showing individual SGLT2 inhibitors comparisons in patients with and without diabetes for outcomes of (A)death, (B) cardiovascular death, (C)cardiovascular death or HHF, (D)kidney progression, (E)AKI, (F)ketoacidosis, (G)lower limbs amputation, (H)UTI, (I)Mycotic genital infection, (J)Hypoglycemia, (K)Bone fracture. **Figure S5.** The complete results of forest plots and SUCRA showing individual SGLT2 inhibitors comparisons in patients with and without CKD for (A) renal-specific outcome and (B) cardiovascular death or HHFand in patients with and without HF for (C) cardiovascular death or HHF and (D) major adverse cardiovascular events. **Figure S6.** The results of forest plots and SUCRA showing comparison of highly selective SGLT2 inhibitors and less selective SGLT2 inhibitors in patients with and without diabetes for outcomes of (A)death, (B) cardiovascular death, (C)cardiovascular death or HHF, (D)kidney progression, (E)AKI, (F)ketoacidosis, (G)lower limbs amputation, (H)UTI, (I)Mycotic genital infection, (J)Hypoglycemia, (K)Bone fracture. **Figure S7.** The results of forest plots and SUCRA depicting highly selective SGLT2 inhibitors vs less selective SGLT2 inhibitors in patients with and without CKD for (A)renal-specific composite outcome and (B)cardiovascular death or in HHF and in patients with and without HF for (C)cardiovascular death or HHF and (D)major adverse cardiovascular events. **Figure S8.** Circular barplot of the main results.

## Data Availability

All data generated or analyzed during this study are included in the published article.

## Abbreviations

AKI: Acute kidney injury
CANVAS: Canagliflozin and cardiovascular and renal events in type 2 diabetes
CKD: Chronic kidney disease
CREDENCE: Canagliflozin and renal outcomes in type 2 diabetes and nephropathy
CV: Cardiovascular
DAPA-CKD: Dapagliflozin in patients with chronic kidney disease
DAPA-HF: Dapagliflozin in patients with heart failure and reduced ejection fraction
DM: Diabetes mellitus
EMPA-KIDNEY: Empagliflozin in patients with chronic kidney disease
EMPEROR-Reduced: Cardiovascular and renal outcomes with empagliflozin in heart failure
EMPA-REG: Empagliflozin, cardiovascular outcomes, and mortality in type 2 diabetes
HF: Heart failure
HHF: Hospitalization for heart failure
HFpEF: Heart failure with preserved ejection fraction
HFrEF: Heart failure with reduced ejection fraction
MACEs: Major adverse cardiac events
NR: Not reported
RCTs: Randomized controlled trials
RRs: Risk ratios
SGLT2i: Sodium-glucose cotransporter 2 inhibitor
SUCRA: Surface under the cumulative ranking
TSA: Trial sequential analysis
UTI: Urinary tract infection

## References

[1] Cowie MR, Fisher M (2020). SGLT2 inhibitors: mechanisms of cardiovascular benefit beyond glycaemic control. Nat Rev Cardiol.

[2] Zinman B, Wanner C, Lachin JM, Fitchett D, Bluhmki E, Hantel S, Mattheus M, Devins T, Johansen OE, Woerle HJ (2015). Empagliflozin, cardiovascular outcomes, and mortality in type 2 diabetes. N Engl J Med.

[3] Neal B, Perkovic V, Mahaffey KW, de Zeeuw D, Fulcher G, Erondu N, Shaw W, Law G, Desai M, Matthews DR (2017). Canagliflozin and cardiovascular and renal events in type 2 diabetes. N Engl J Med.

[4] Wiviott SD, Raz I, Bonaca MP, Mosenzon O, Kato ET, Cahn A, Silverman MG, Zelniker TA, Kuder JF, Murphy SA (2019). Dapagliflozin and cardiovascular outcomes in type 2 diabetes. N Engl J Med.

[5] McMurray JJV, Solomon SD, Inzucchi SE, Køber L, Kosiborod MN, Martinez FA, Ponikowski P, Sabatine MS, Anand IS, Bělohlávek J (2019). Dapagliflozin in patients with heart failure and reduced ejection fraction. N Engl J Med.

[6] Packer M, Anker SD, Butler J, Filippatos G, Pocock SJ, Carson P, Januzzi J, Verma S, Tsutsui H, Brueckmann M (2020). Cardiovascular and renal outcomes with empagliflozin in heart failure. N Engl J Med.

[7] Perkovic V, Jardine MJ, Neal B, Bompoint S, Heerspink HJL, Charytan DM, Edwards R, Agarwal R, Bakris G, Bull S (2019). Canagliflozin and renal outcomes in type 2 diabetes and nephropathy. N Engl J Med.

[8] Heerspink HJL, Stefánsson BV, Correa-Rotter R, Chertow GM, Greene T, Hou FF, Mann JFE, McMurray JJV, Lindberg M, Rossing P (2020). Dapagliflozin in patients with chronic kidney disease. N Engl J Med.

[9] Herrington WG, Staplin N, Wanner C, Green JB, Hauske SJ, Emberson JR, Preiss D, Judge P, Mayne KJ, Group E-KC (2022). Empagliflozin in patients with chronic kidney disease. N Engl J Med.

[10] Täger T, Frankenstein L, Atar D, Agewall S, Frey N, Grundtvig M, Clark AL, Cleland JG, Fröhlich H (2022). Influence of receptor selectivity on benefits from SGLT2 inhibitors in patients with heart failure: a systematic review and head-to-head comparative efficacy network meta-analysis. Clin Res Cardiol.

[11] Takebayashi K, Inukai T (2017). Effect of sodium glucose cotransporter 2 inhibitors with low SGLT2/sglt1 selectivity on circulating glucagon-like peptide 1 levels in type 2 diabetes mellitus. J Clin Med Res.

[12] Zhou L, Cryan EV, D'Andrea MR, Belkowski S, Conway BR, Demarest KT (2003). Human cardiomyocytes express high level of Na+/glucose cotransporter 1 (SGLT1). J Cell Biochem.

[13] Toyama T, Neuen BL, Jun M, Ohkuma T, Neal B, Jardine MJ, Heerspink HL, Wong MG, Ninomiya T, Wada T (2019). Effect of SGLT2 inhibitors on cardiovascular, renal and safety outcomes in patients with type 2 diabetes mellitus and chronic kidney disease: a systematic review and meta-analysis. Diabetes Obes Metab.

[14] Zelniker TA, Wiviott SD, Raz I, Im K, Goodrich EL, Bonaca MP, Mosenzon O, Kato ET, Cahn A, Furtado RH (2019). SGLT2 inhibitors for primary and secondary prevention of cardiovascular and renal outcomes in type 2 diabetes: a systematic review and meta-analysis of cardiovascular outcome trials. Lancet.

[15] Zannad F, Ferreira JP, Pocock SJ, Anker SD, Butler J, Filippatos G, Brueckmann M, Ofstad AP, Pfarr E, Jamal W (2020). SGLT2 inhibitors in patients with heart failure with reduced ejection fraction: a meta-analysis of the EMPEROR-Reduced and DAPA-HF trials. Lancet.

[16] Lo KB, Gul F, Ram P, Kluger AY, Tecson KM, McCullough PA, Rangaswami J (2020). The effects of SGLT2 inhibitors on cardiovascular and renal outcomes in diabetic patients: a systematic review and meta-analysis. Cardiorenal Med.

[17] McGuire DK, Shih WJ, Cosentino F, Charbonnel B, Cherney DZI, Dagogo-Jack S, Pratley R, Greenberg M, Wang S, Huyck S (2021). Association of SGLT2 inhibitors with cardiovascular and kidney outcomes in patients with type 2 diabetes: a meta-analysis. JAMA Cardiol.

[18] Martínez-Vizcaíno V, Díez-Fernández A, Álvarez-Bueno C, Martínez-Alfonso J, Cavero-Redondo I (2021). Safety and efficacy of sglt2 inhibitors: a multiple-treatment meta-analysis of clinical decision indicators. J Clin Med.

[19] Baigent C, Emberson J, Haynes R, Herrington WG, Judge P, Landray MJ, Mayne KJ, Ng SY, Preiss D, Roddick AJ, Staplin N (2022). Impact of diabetes on the effects of sodium glucose co-transporter-2 inhibitors on kidney outcomes: collaborative meta-analysis of large placebo-controlled trials. Lancet.

[20] Voors AA, Angermann CE, Teerlink JR, Collins SP, Kosiborod M, Biegus J, Ferreira JP, Nassif ME, Psotka MA, Tromp J (2022). The SGLT2 inhibitor empagliflozin in patients hospitalized for acute heart failure: a multinational randomized trial. Nat Med.

[21] Higgins JP, Altman DG, Gøtzsche PC, Jüni P, Moher D, Oxman AD, Savovic J, Schulz KF, Weeks L, Sterne JA (2011). The Cochrane Collaboration's tool for assessing risk of bias in randomised trials. BMJ.

[22] Salguero G, Akin E, Templin C, Kotlarz D, Doerries C, Landmesser U, Grote K, Schieffer B (2008). Renovascular hypertension by two-kidney one-clip enhances endothelial progenitor cell mobilization in a p47phox-dependent manner. J Hypertens.

[23] Owen RK, Bradbury N, Xin Y, Cooper N, Sutton A (2019). MetaInsight: An interactive web-based tool for analyzing, interrogating, and visualizing network meta-analyses using R-shiny and netmeta. Res Synth Methods.

[24] Salanti G, Ades AE, Ioannidis JP (2011). Graphical methods and numerical summaries for presenting results from multiple-treatment meta-analysis: an overview and tutorial. J Clin Epidemiol.

[25] Nikolakopoulou A, Mavridis D, Egger M, Salanti G (2018). Continuously updated network meta-analysis and statistical monitoring for timely decision-making. Stat Methods Med Res.

[26] Chaimani A, Higgins JP, Mavridis D, Spyridonos P, Salanti G (2013). Graphical tools for network meta-analysis in STATA. PLoS ONE.

[27] Anker SD, Butler J, Filippatos G, Ferreira JP, Bocchi E, Böhm M, Brunner-La Rocca HP, Choi DJ, Chopra V, Chuquiure-Valenzuela E (2021). Empagliflozin in heart failure with a preserved ejection fraction. N Engl J Med.

[28] Petrie MC, Verma S, Docherty KF, Inzucchi SE, Anand I, Belohlávek J, Böhm M, Chiang CE, Chopra VK, de Boer RA (2020). Effect of dapagliflozin on worsening heart failure and cardiovascular death in patients with heart failure with and without diabetes. JAMA.

[29] Solomon SD, McMurray JJV, Claggett B, de Boer RA, DeMets D, Hernandez AF, Inzucchi SE, Kosiborod MN, Lam CSP, Martinez F (2022). Dapagliflozin in heart failure with mildly reduced or preserved ejection fraction. N Engl J Med.

[30] Heerspink HJ, Perkins BA, Fitchett DH, Husain M, Cherney DZ (2016). Sodium glucose cotransporter 2 inhibitors in the treatment of diabetes mellitus: cardiovascular and kidney effects, potential mechanisms, and clinical applications. Circulation.

[31] Del Vecchio L, Beretta A, Jovane C, Peiti S, Genovesi S (2021). A role for SGLT-2 inhibitors in treating non-diabetic chronic kidney disease. Drugs.

[32] Chen S, Coronel R, Hollmann MW, Weber NC, Zuurbier CJ (2022). Direct cardiac effects of SGLT2 inhibitors. Cardiovasc Diabetol.

[33] Zou R, Shi W, Qiu J, Zhou N, Du N, Zhou H, Chen X, Ma L (2022). Empagliflozin attenuates cardiac microvascular ischemia/reperfusion injury through improving mitochondrial homeostasis. Cardiovasc Diabetol.

[34] Mone P, Varzideh F, Jankauskas SS, Pansini A, Lombardi A, Frullone S, Santulli G (2022). SGLT2 inhibition via empagliflozin improves endothelial function and reduces mitochondrial oxidative stress: insights from frail hypertensive and diabetic patients. Hypertension.

[35] Santulli G, Varzideh F, Forzano I, Wilson S, Salemme L, de Donato A, Lombardi A, Rainone A, Nunziata L, Jankauskas SS (2023). Functional and clinical importance of sglt2-inhibitors in frailty: from the kidney to the heart. Hypertension.

[36] Paolisso P, Bergamaschi L, Santulli G, Gallinoro E, Cesaro A, Gragnano F, Sardu C, Mileva N, Foà A, Armillotta M (2022). Infarct size, inflammatory burden, and admission hyperglycemia in diabetic patients with acute myocardial infarction treated with SGLT2-inhibitors: a multicenter international registry. Cardiovasc Diabetol.

[37] Patorno E, Pawar A, Wexler DJ, Glynn RJ, Bessette LG, Paik JM, Najafzadeh M, Brodovicz KG, Déruaz-Luyet A, Schneeweiss S (2022). Effectiveness and safety of empagliflozin in routine care patients: results from the EMPagliflozin compaRative effectIveness and SafEty (EMPRISE) study. Diabetes Obes Metab.

[38] Palmer SC, Tendal B, Mustafa RA, Vandvik PO, Li S, Hao Q, Tunnicliffe D, Ruospo M, Natale P, Saglimbene V (2021). Sodium-glucose cotransporter protein-2 (SGLT-2) inhibitors and glucagon-like peptide-1 (GLP-1) receptor agonists for type 2 diabetes: systematic review and network meta-analysis of randomised controlled trials. BMJ.

[39] Demin O, Yakovleva T, Kolobkov D, Demin O (2014). Analysis of the efficacy of SGLT2 inhibitors using semi-mechanistic model. Front Pharmacol.

[40] Vrhovac I, Balen Eror D, Klessen D, Burger C, Breljak D, Kraus O, Radović N, Jadrijević S, Aleksic I, Walles T (2015). Localizations of Na+-D-glucose cotransporters SGLT1 and SGLT2 in human kidney and of SGLT1 in human small intestine, liver, lung, and heart. Pflügers Archiv-Eur J Physiol.

[41] Kashiwagi Y, Nagoshi T, Yoshino T, Tanaka TD, Ito K, Harada T, Takahashi H, Ikegami M, Anzawa R, Yoshimura M (2015). Expression of SGLT1 in human hearts and impairment of cardiac glucose uptake by phlorizin during ischemia-reperfusion injury in mice. PLoS ONE.

[42] Yoshii A, Nagoshi T, Kashiwagi Y, Kimura H, Tanaka Y, Oi Y, Ito K, Yoshino T, Tanaka TD, Yoshimura M (2019). Cardiac ischemia–reperfusion injury under insulin-resistant conditions: SGLT1 but not SGLT2 plays a compensatory protective role in diet-induced obesity. Cardiovasc Diabetol.

[43] Fortin E, Lundin M, Mellbin L, Norhammar A, Näsman P, Smetana S, Sörensson P, Ferrannini E, Rydén L, Ferrannini G (2023). Empagliflozin improves insulin sensitivity in patients with recent acute coronary syndrome and newly detected dysglycaemia : Experiences from the randomized, controlled SOCOGAMI trial. Cardiovasc Diabetol.

[44] Mone P, Lombardi A, Kansakar U, Varzideh F, Jankauskas SS, Pansini A, Marzocco S, De Gennaro S, Famiglietti M, Macina G (2023). Empagliflozin improves the MicroRNA signature of endothelial dysfunction in patients with heart failure with preserved ejection fraction and diabetes. J Pharmacol Exp Ther.

[45] Carluccio E, Biagioli P, Reboldi G, Mengoni A, Lauciello R, Zuchi C, D'Addario S, Bardelli G, Ambrosio G (2023). Left ventricular remodeling response to SGLT2 inhibitors in heart failure: an updated meta-analysis of randomized controlled studies. Cardiovasc Diabetol.

[46] von Lewinski D, Kolesnik E, Tripolt NJ, Pferschy PN, Benedikt M, Wallner M, Alber H, Berger R, Lichtenauer M, Saely CH (2022). Empagliflozin in acute myocardial infarction: the EMMY trial. Eur Heart J.

[47] Barrera-Chimal J, Jaisser F (2020). Pathophysiologic mechanisms in diabetic kidney disease: a focus on current and future therapeutic targets. Diabetes Obes Metab.

[48] Bhatt DL, Szarek M, Pitt B, Cannon CP, Leiter LA, McGuire DK, Lewis JB, Riddle MC, Inzucchi SE, Kosiborod MN (2021). Sotagliflozin in patients with diabetes and chronic kidney disease. N Engl J Med.

[49] Bhatt DL, Szarek M, Steg PG, Cannon CP, Leiter LA, McGuire DK, Lewis JB, Riddle MC, Voors AA, Metra M (2021). Sotagliflozin in patients with diabetes and recent worsening heart failure. N Engl J Med.

[50] Vrhovac I, Balen Eror D, Klessen D, Burger C, Breljak D, Kraus O, Radović N, Jadrijević S, Aleksic I, Walles T (2015). Localizations of Na(+)-D-glucose cotransporters SGLT1 and SGLT2 in human kidney and of SGLT1 in human small intestine, liver, lung, and heart. Pflugers Arch.

[51] McNeill AM, Davies G, Kruger E, Kowal S, Reason T, Ejzykowicz F, Hannachi H, Cater N, McLeod E (2019). Ertugliflozin compared to other anti-hyperglycemic agents as monotherapy and add-on therapy in type 2 diabetes: a systematic literature review and network meta-analysis. Diabetes Therapy.

[52] Liu L, Shi F-H, Xu H, Wu Y, Gu Z-C, Lin H-W (2022). Efficacy and safety of ertugliflozin in type 2 diabetes: a systematic review and meta-analysis. Front Pharmacol.

[53] Lin C, Zhu X, Cai X, Yang W, Lv F, Nie L, Ji L (2021). SGLT2 inhibitors and lower limb complications: an updated meta-analysis. Cardiovasc Diabetol.

[54] Erkens JA, Klungel OH, Stolk RP, Spoelstra JA, Grobbee DE, Leufkens HG (2004). Antihypertensive drug therapy and the risk of lower extremity amputations in pharmacologically treated type 2 diabetes patients. Pharmacoepidemiol Drug Saf.

[55] Guyatt GH, Oxman AD, Kunz R, Woodcock J, Brozek J, Helfand M, Alonso-Coello P, Glasziou P, Jaeschke R, Akl EA (2011). GRADE guidelines: 7. Rating the quality of evidence—inconsistency. J Clin Epidemiol.

[56] Savarese G, Stolfo D, Sinagra G, Lund LH (2022). Heart failure with mid-range or mildly reduced ejection fraction. Nat Rev Cardiol.

[57] Fernández-Balsells MM, Sojo-Vega L, Ricart-Engel W (2017). Canagliflozin and cardiovascular and renal events in type 2 diabetes. N Engl J Med.

[58] Furtado RHM, Bonaca MP, Raz I, Zelniker TA, Mosenzon O, Cahn A, Kuder J, Murphy SA, Bhatt DL, Leiter LA (2019). Dapagliflozin and cardiovascular outcomes in patients with type 2 diabetes mellitus and previous myocardial infarction. Circulation.

[59] Cannon CP, Pratley R, Dagogo-Jack S, Mancuso J, Huyck S, Masiukiewicz U, Charbonnel B, Frederich R, Gallo S, Cosentino F (2020). Cardiovascular outcomes with ertugliflozin in type 2 diabetes. N Engl J Med.

