# Supplementary material for: Impact of SGLT2 inhibitors on patient outcomes: a network meta-analysis
Source: Cardiovasc Diabetol. 2023 Oct 27;22:290. doi: 10.1186/s12933-023-02035-8 (PMC10612254; doi:10.1186/s12933-023-02035-8)
Supplement: Supplementary file 1 — Additional file 1: Figure S1. Network geometry and forest plot of selected results, including (A)DM patients with death; (B)non-DM patients with cardiovascular death or HHF; (C)Non-DM patients with AKI; (D)CKD patients with cardiovascular death or HHF and (E)non-CKD patients with cardiovascular death or HHF. Figure S2. Areas under the cumulative ranking curves for of selected results, including individual SGLT2 inhibitors with regard to (A) death, (B) AKI among DM patients, (C) AKI among non-DM patients, (D) cardiovascular death or HHF among HF patients, (E) cardiovascular death or HHF among non-HF patients, (F) MACE among HF patients (G) MACE among HF patients for highly selective SGLT2 inhibitors (dapagliflozin, empagliflozin, ertugliflozin) and less selective SGLT2 inhibitors (canagliflozin, sotagliflozin). Figure S3. Sequential network meta-analyses of selected results, including (A)Mortality among DM/non-DM, (B) AKI for DM/non-DM, (C) MACE for HF/non-HF patients with SGLT2 inhibitors versus placebo. Figure S4. The complete results of forest plots and SUCRA showing individual SGLT2 inhibitors comparisons in patients with and without diabetes for outcomes of (A)death, (B) cardiovascular death, (C)cardiovascular death or HHF, (D)kidney progression, (E)AKI, (F)ketoacidosis, (G)lower limbs amputation, (H)UTI, (I)Mycotic genital infection, (J)Hypoglycemia, (K)Bone fracture. Figure S5. The complete results of forest plots and SUCRA showing individual SGLT2 inhibitors comparisons in patients with and without CKD for (A) renal-specific outcome and (B) cardiovascular death or HHFand in patients with and without HF for (C) cardiovascular death or HHF and (D) major adverse cardiovascular events. Figure S6. The results of forest plots and SUCRA showing comparison of highly selective SGLT2 inhibitors and less selective SGLT2 inhibitors in patients with and without diabetes for outcomes of (A)death, (B) cardiovascular death, (C)cardiovascular death or HHF, (D)kidney progressi [file 12933_2023_2035_MOESM1_ESM.docx]

**Impact of SGLT2 Inhibitors on Patient Outcomes: A Network Meta-analysis**

**Additional appendix**

Authors: Jui-Yi Chen, Heng-Chih Pan, Chih-Chung Shiao, Min-Hsiang Chuang, Chun Yin See, Tzu-Hsuan Yeh, Yafei Yang, Wen-Kai Chu, Vin-Cent Wu

This Additional appendix provides:

1. Search equation via PubMed, EMBASE and [Cochrane](http://www.cochranelibrary.com/) library
2. Risk of bias 2.0
3. Additional Figures

**Additional Figure 1.** Network geometry and forest plot of selected results, including (A)DM patients with death; (B)non-DM patients with cardiovascular death or HHF; (C)Non-DM patients with AKI; (D)CKD patients with cardiovascular death or HHF and (E)non-CKD patients with cardiovascular death or HHF

**Additional Figure 2.** Areas under the cumulative ranking curves for of selected results, including individual SGLT2 inhibitors with regard to (A) death, (B) AKI among DM patients, (C) AKI among non-DM patients, (D) cardiovascular death or HHF among HF patients, (E) cardiovascular death or HHF among non-HF patients, (F) MACE among HF patients (G) MACE among HF patients for highly selective SGLT2 inhibitors (dapagliflozin, empagliflozin, ertugliflozin) and less selective SGLT2 inhibitors (canagliflozin, sotagliflozin)

**Additional Figure 3.** Sequential network meta-analyses of selected results, including (A)Mortality among DM/non-DM, (B) AKI for DM/non-DM, (C) MACE for HF/non-HF patients with SGLT2 inhibitors versus placebo

**Additional Figure 4.** The complete results of forest plots and SUCRA showing individual SGLT2 inhibitors comparisons in patients with and without diabetes for outcomes of (A)death, (B) cardiovascular death, (C)cardiovascular death or HHF, (D)kidney progression, (E)AKI, (F)ketoacidosis, (G)lower limbs amputation, (H)UTI, (I)Mycotic genital infection, (J)Hypoglycemia, (K)Bone fracture.

**Additional Figure 5.** The complete results of forest plots and SUCRA showing individual SGLT2 inhibitors comparisons in patients with and without CKD for (A) renal-specific outcome and (B) cardiovascular death or HHFand in patients with and without HF for (C) cardiovascular death or HHF and (D) major adverse cardiovascular events.

**Additional Figure 6.** The results of forest plots and SUCRA showing comparison of highly selective SGLT2 inhibitors and less selective SGLT2 inhibitors in patients with and without diabetes for outcomes of (A)death, (B) cardiovascular death, (C)cardiovascular death or HHF, (D)kidney progression, (E)AKI, (F)ketoacidosis, (G)lower limbs amputation, (H)UTI, (I)Mycotic genital infection, (J)Hypoglycemia, (K)Bone fracture

**Additional Figure 7.** The results of forest plots and SUCRA depicting highly selective SGLT2 inhibitors vs less selective SGLT2 inhibitors in patients with and without CKD for (A)renal-specific composite outcome and (B)cardiovascular death or in HHF and in patients with and without HF for (C)cardiovascular death or HHF and (D)major adverse cardiovascular events

**Additional Figure 8.** Circular barplot of the main results

1. The detailed results of all possible comparisons
2. Funnel plot for publication bias
3. Assessing confidence of evidence according to CINeMA.
4. PROSPERO protocol registration.
5. Summary of the feature in each study
6. PRISMA checklist.

**1. Search equation via PubMed, EMBASE, and [Cochrane](http://www.cochranelibrary.com/) library**

**Appendix.**

Search strategies for the different databases ran on March 28, 2023.

**PubMed Search Query**

((((((((((((Sodium glucose cotransporter 2 inhibitor) OR (SGLT2 inhibitors)) OR (canagliflozin)) OR (dapagliflozin)) OR (empagliflozin)) OR (ipragliflozin)) OR (tofogliflozin)) OR (luseogliflozin)) OR (sergliflozin)) OR (remogliflozin)) OR (ertugliflozin)) OR (sotagliflozin)) AND (((mortality) OR (death)) OR (cardiovascular death))

((("sodium glucose transport proteins"[MeSH Terms] OR ("sodium glucose"[All Fields] AND "transport"[All Fields] AND "proteins"[All Fields]) OR "sodium glucose transport proteins"[All Fields] OR ("sodium"[All Fields] AND "glucose"[All Fields] AND "cotransporter"[All Fields]) OR "sodium glucose cotransporter"[All Fields]) AND "2"[All Fields] AND ("antagonists and inhibitors"[MeSH Subheading] OR ("antagonists"[All Fields] AND "inhibitors"[All Fields]) OR "antagonists and inhibitors"[All Fields] OR "inhibitors"[All Fields] OR "inhibitor"[All Fields] OR "inhibitor s"[All Fields])) OR ("sodium glucose transporter 2 inhibitors"[Pharmacological Action] OR "sodium glucose transporter 2 inhibitors"[MeSH Terms] OR "sodium glucose transporter 2 inhibitors"[All Fields] OR ("sglt2"[All Fields] AND "inhibitors"[All Fields]) OR "sglt2 inhibitors"[All Fields]) OR ("canagliflozin"[MeSH Terms] OR "canagliflozin"[All Fields]) OR ("dapagliflozin"[Additional Concept] OR "dapagliflozin"[All Fields] OR "dapagliflozin s"[All Fields]) OR ("empagliflozin"[Additional Concept] OR "empagliflozin"[All Fields]) OR ("ipragliflozin"[Additional Concept] OR "ipragliflozin"[All Fields]) OR ("6 4 ethylphenyl methyl 3 4 5 6 tetrahydro 6 hydroxymethyl spiro isobenzofuran 1 3h 2 2h pyran 3 4 5 triol"[Additional Concept] OR "6 4 ethylphenyl methyl 3 4 5 6 tetrahydro 6 hydroxymethyl spiro isobenzofuran 1 3h 2 2h pyran 3 4 5 triol"[All Fields] OR "tofogliflozin"[All Fields]) OR ("1 5 anhydro 1 5 4 ethoxybenzyl 2 methoxy 4 methylphenyl 1 thioglucitol"[Additional Concept] OR "1 5 anhydro 1 5 4 ethoxybenzyl 2 methoxy 4 methylphenyl 1 thioglucitol"[All Fields] OR "luseogliflozin"[All Fields]) OR ("sergliflozin"[Additional Concept] OR "sergliflozin"[All Fields]) OR "remogliflozin"[All Fields] OR ("ertugliflozin"[Additional Concept] OR "ertugliflozin"[All Fields]) OR ("2s 3r 4r 5s 6r 2 4 chloro 3 4 ethoxybenzyl phenyl 6 methylthio tetrahydro 2h pyran 3 4 5 triol"[Additional Concept] OR "2s 3r 4r 5s 6r 2 4 chloro 3 4 ethoxybenzyl phenyl 6 methylthio tetrahydro 2h pyran 3 4 5 triol"[All Fields] OR "sotagliflozin"[All Fields])) AND ("mortality"[MeSH Terms] OR "mortality"[All Fields] OR "mortalities"[All Fields] OR "mortality"[MeSH Subheading] OR ("death"[MeSH Terms] OR "death"[All Fields] OR "deaths"[All Fields]) OR (("cardiovascular system"[MeSH Terms] OR ("cardiovascular"[All Fields] AND "system"[All Fields]) OR "cardiovascular system"[All Fields] OR "cardiovascular"[All Fields] OR "cardiovasculars"[All Fields]) AND ("death"[MeSH Terms] OR "death"[All Fields] OR "deaths"[All Fields])))

**EMBASE**

No. Query Results

#5 #4 AND 'randomized controlled trial topic'/de 456

#4 #1 AND #2 AND #3 2794

#3 'heart failure'/exp OR 'heart failure' 685918

#2 'mortality'/exp OR mortality OR 'death'/exp OR 'cardiovascular death'/exp 2428017

#1 'sodium glucose cotransporter 2 inhibitor'/exp OR 'sodium glucose cotransporter 2 inhibitor' OR 'canagliflozin'/exp OR 'dapagliflozin'/exp OR 'empagliflozin'/exp OR 'ipragliflozin'/exp OR 'tofogliflozin'/exp OR 'luseogliflozin'/exp OR 'sergliflozin etabonate'/exp OR 'remogliflozin'/exp OR 'ertugliflozin'/exp OR 'sotagliflozin'/exp 20238

**Cochrane Library**

ID Search Hits

#1 MeSH descriptor: [Sodium-Glucose Transporter 2 Inhibitors] explode all trees 543

#2 dapagliflozin 1582

#3 empagliflozin 1457

#4 canagliflozin 685

#5 ipragliflozin 164

#6 tofogliflozin 95

#7 luseogliflozin 101

#8 sergliflozin 6

#9 remogliflozin 34

#10 ertugliflozin 175

#11 sotagliflozin 115

#12 #1 or #2 or #3 or #4 or #5 or #6 or #7 or #8 or #9 or #10 or #11 4177

#13 mortality 109178

#14 death 77006

#15 cardiovascular death 17813

#16 #13 or # 14 or # 15 579062

#17 #12 AND #16 1755

#18 heart failure 43789

#19 #16 AND # 18 157634

#20 #12 AND #19 544

**2. Risk of bias assessment using version 2 of Cochrane risk-of-bias tool for randomized trials (RoB 2)**

**
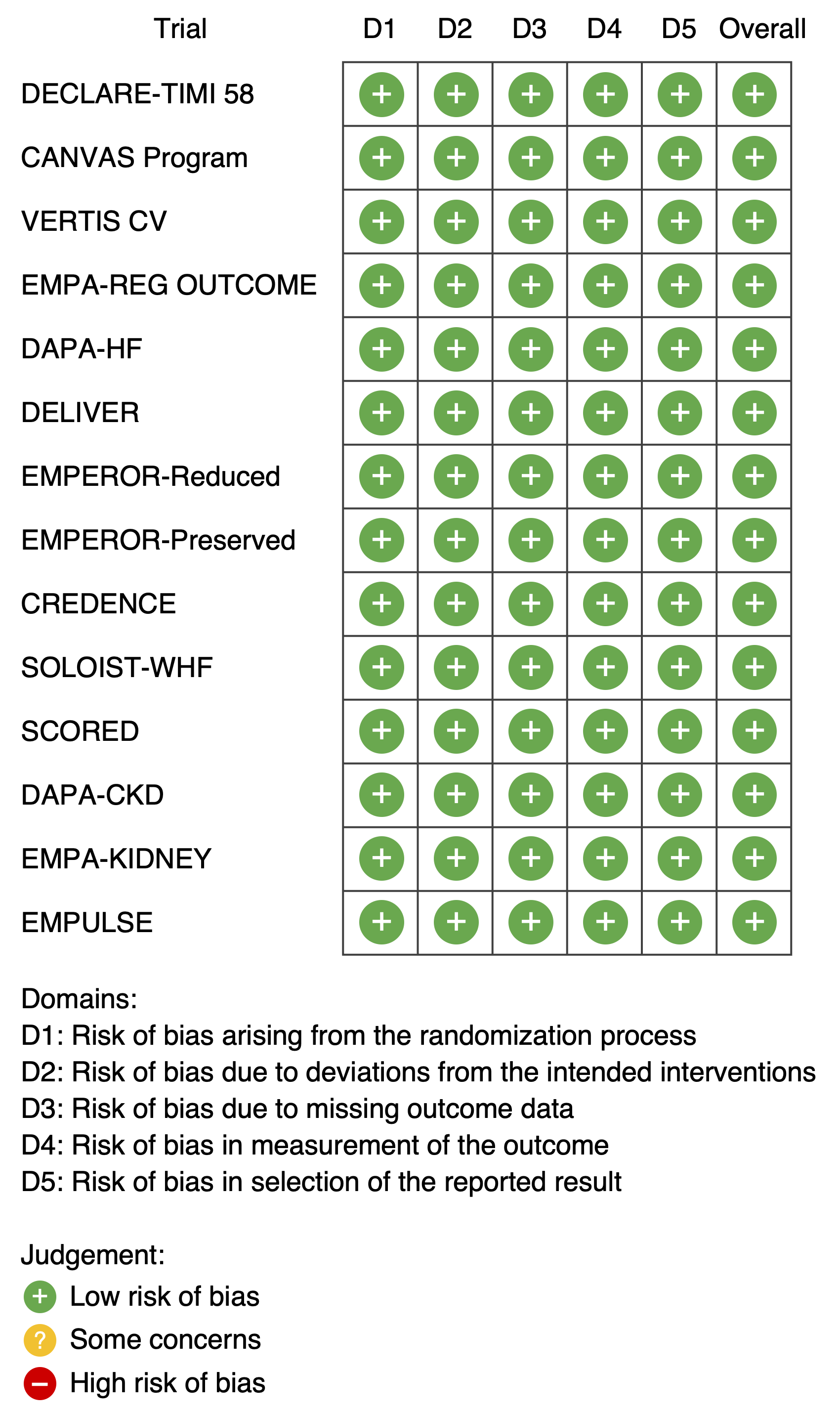
**

**3. Additional Figures**

**Additional Figure 1.** Network geometry and forest plot of selected results, including (A)DM patients with death; (B)non-DM patients with cardiovascular death or HHF; (C)Non-DM patients with AKI; (D)CKD patients with cardiovascular death or HHF and (E)non-CKD patients with cardiovascular death or HHF


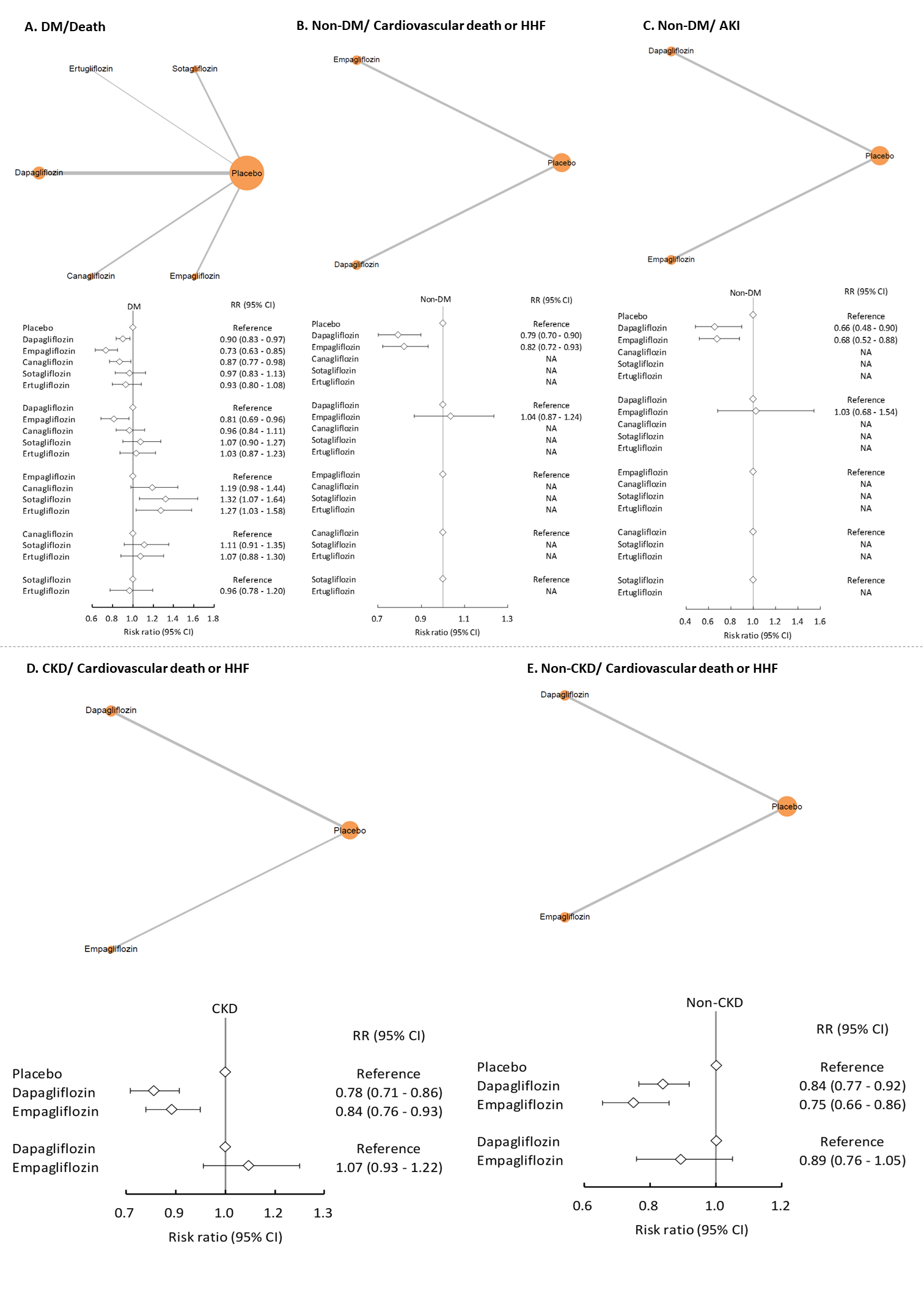


**Abbreviations:** AKI, acute kidney injury; CKD, Chronic kidney disease; DM, Diabetes Mellitus; HF, Heart failure; HHF, hospitalization heart failure; MACE, Major adverse cardiac events

**Additional Figure 2**. Areas under the cumulative ranking curves for of selected results, including individual SGLT2 inhibitors with regard to (A) death, (B) AKI among DM patients, (C) AKI among non-DM patients, (D) cardiovascular death or HHF among HF patients, (E) cardiovascular death or HHF among non-HF patients, (F) MACE among HF patients and (G) MACE among HF patients for highly selective SGLT2 inhibitors (dapagliflozin, empagliflozin, ertugliflozin) and less selective SGLT2 inhibitors (canagliflozin, sotagliflozin)

**Abbreviations**: AKI, acute kidney injury; DM, Diabetes Mellitus; HF, Heart failure; HHF: Hospitalization heart failure; MACE: Major adverse cardiac events; SUCRA: Surface under the cumulative ranking curve area

Dark color bar represents significantly higher values.

**
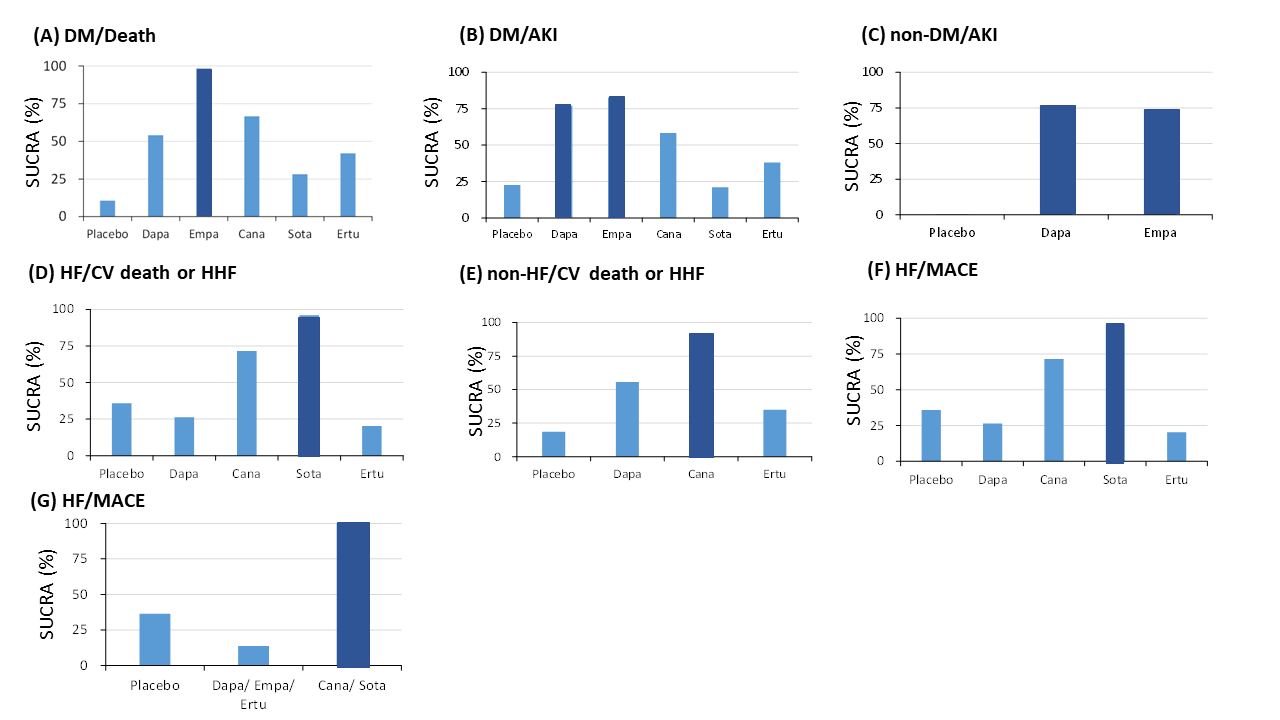
**

**Additional Figure 3.** Sequential network meta-analyses of selected results, including (A)Mortality among DM/non-DM, (B)AKI for DM/non-DM, (C)MACE for HF/non-HF patients with SGLT2 inhibitors versus placebo

**Abbreviations:** AKI, Acute kidney injury; DM, Diabetes Mellitus; HF, Heart failure; MACE, Major adverse cardiac events; SGLT2, Sodium–Glucose Cotransporter 2


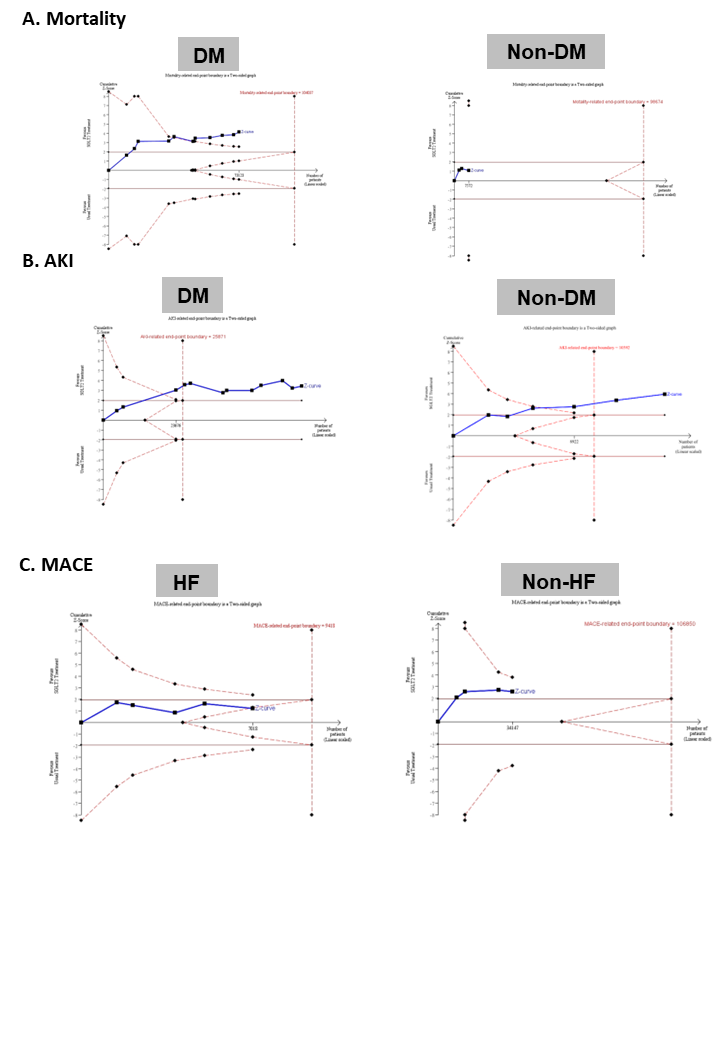


**Additional Figure 4.** The complete results of forest plots and SUCRA showing individual SGLT2 inhibitors comparisons in patients with and without diabetes for outcomes of (A)death, (B) cardiovascular death, (C)cardiovascular death or HHF, (D)kidney progression, (E)AKI, (F)ketoacidosis, (G)lower limbs amputation, (H)UTI, (I)Mycotic genital infection, (J)Hypoglycemia, (K)Bone fracture.

|  |  |
| --- | --- |
|  |  |
|  |  |
|  |  |
|  |  |
|  |  |

**Additional Figure 5.** The complete results of forest plots and SUCRA showing individual SGLT2 inhibitors comparisons in patients with and without CKD for (A) renal-specific outcome and (B) cardiovascular death or HHF and in patients with and without HF for (C) cardiovascular death or HHF and (D) major adverse cardiovascular events.

**Abbreviations:** AKI, Acute kidney injury; DM, Diabetes mellitus; HHF, Hospitalization heart failure; SUCRA, surface under the cumulative ranking curve area

| **** | **** |
| --- | --- |
| **** | **** |

**Additional Figure 6.** The results of forest plots and SUCRA showing comparison of highly selective SGLT2 inhibitors and less selective SGLT2 inhibitors in patients with and without diabetes for outcomes of (A)death, (B) cardiovascular death, (C)cardiovascular death or HHF, (D)kidney progression, (E)AKI, (F)ketoacidosis, (G)lower limbs amputation, (H)UTI, (I)Mycotic genital infection, (J)Hypoglycemia, (K)Bone fracture

**Abbreviations:** AKI, Acute kidney injury; DM, Diabetes mellitus; HHF, Hospitalization heart failure; SUCRA, surface under the cumulative ranking curve area; UTI, Urinary tract infection

| **** | **** |
| --- | --- |
| **** | **** |
| **** | **** |
| **** | **** |
| **** | **** |
| **** |  |

**Additional Figure 7.** The results of forest plots and SUCRA depicting highly selective SGLT2 inhibitors vs less selective SGLT2 inhibitors in patients with and without CKD for (A)renal-specific composite outcome and (B)cardiovascular death or in HHF and in patients with and without HF for (C)cardiovascular death or HHF and (D)major adverse cardiovascular events

**Abbreviations:** CKD, Chronic kidney disease; HHF, Hospitalization heart failure; HF, Heart failure; SUCRA, surface under the cumulative ranking curve area

|  |  |
| --- | --- |
|  |  |

**Additional Figure 8.** Circular barplot of the main results

**
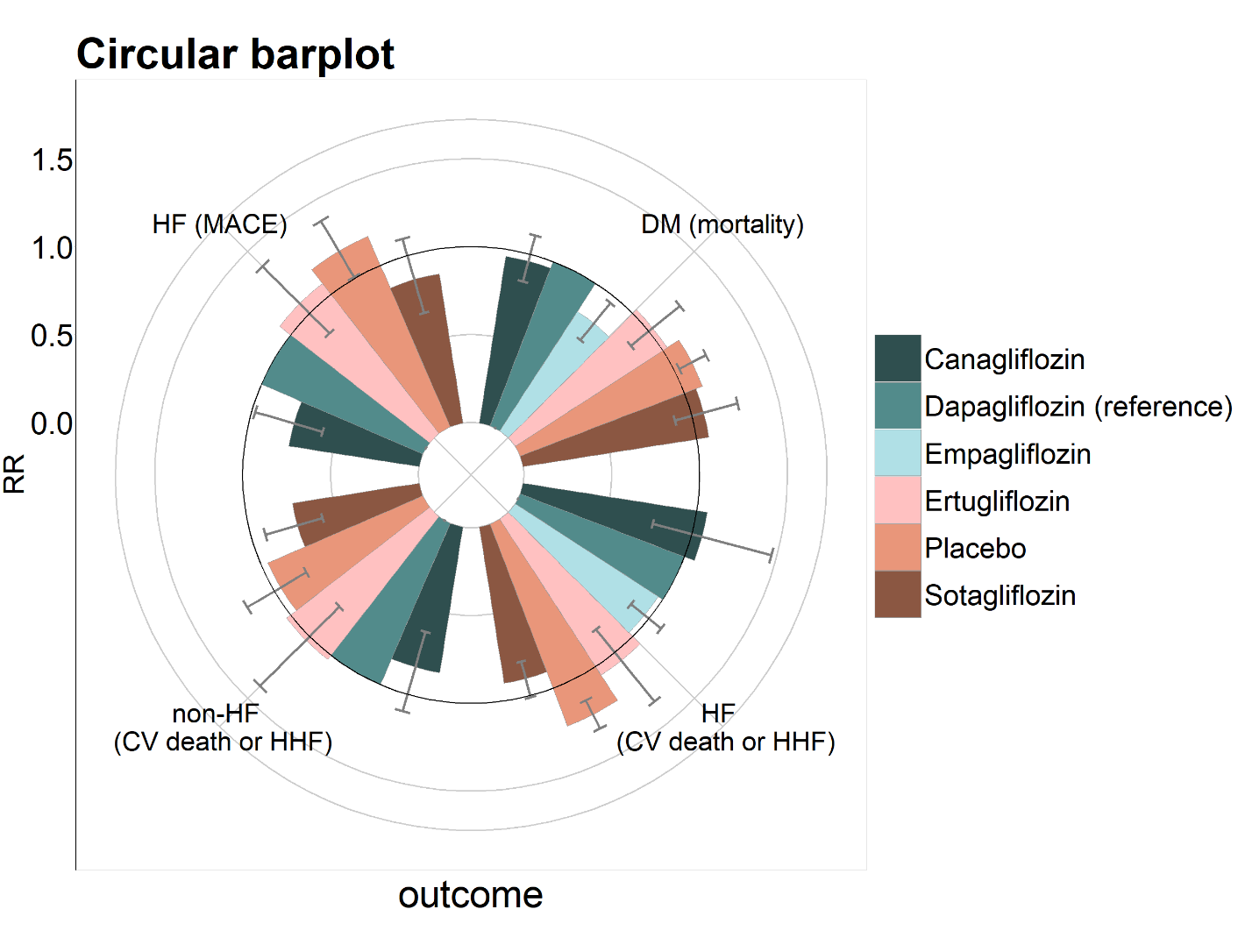
**

**4. The detailed results of all possible comparisons**

**4.1. Results by the presence or absence of DM**

**4.1.1. Death**

A total of 73,123 patients with DM were retrieved from 11 studies, of whom 5,724 died during follow-up (mortality rate 8.0%). Dapagliflozin users (RR: 0.90, 95% CI: 0.83-0.97), empagliflozin users (RR: 0.73, 95% CI: 0.63-0.85), and canagliflozin users (RR: 0.87, 95% CI: 0.77-0.98) were found to have lower risks of death than placebo users (**Additional Figure 1A**). Empagliflozin users had a significantly lower risk of death compared to dapagliflozin users (RR: 0.81, 95% CI: 0.69-0.96) (**Figure 2A, 3A**). SUCRA showed that empagliflozin was associated with a lower risk of death than dapagliflozin (**Additional Figure 2A)**. TSA showed that the cumulative z curve reached the benefit boundary without attaining required information size (**Additional Figure 3A**). Detailed comparisons in the DM and non-DM groups were provided in the supplements (**Additional Figure 4A**).

**4.1.2. CV death or HHF**

A total of 74,804 patients with DM were retrieved from 13 studies, of whom 6,889 developed the composite outcome of CV death or HHF. The incidence rate of CV death or HHF was 9.2%. Compared to placebo users, dapagliflozin users (RR: 0.80, 95% CI: 0.73–0.89), empagliflozin users (RR: 0.76, 95% CI: 0.68–0.88), canagliflozin users (RR: 0.83, 95% CI: 0.72–0.96), and sotagliflozin users (RR: 0.74, 95% CI: 0.65–0.84) were found to have significantly lower risks of CV death or HHF (**Figure 3A, Additional Figure 4B**). Sequential TSA crossed the benefit boundary with required information size (**Figure 4A**). The non-DM patients taking dapagliflozin (RR: 0.79, 95% CI: 0.70-0.90) or empagliflozin (RR: 0.82, CI: 0.72-0.93) had significantly lower risks of cardiovascular death or HHF (**Figure 3A, 4A, and Additional Figure 1B**). Detailed comparisons in the DM and non-DM groups were provided in the supplements (**Additional Figure 4C**).

**4.1.3. Kidney function progression**

A total of 73,530 patients with DM were retrieved from 12 studies, of whom 1,759 had the composite outcome of kidney function progression during follow-up. The incidence rate of kidney function progression was 2.0%. Compared to placebo users, dapagliflozin users (RR: 0.62, 95% CI: 0.53–0.74), empagliflozin users (RR: 0.63, 95% CI: 0.53–0.75), and canagliflozin users (RR: 0.69, 95% CI: 0.58–0.81) had significantly lower risks of kidney progression (**Figure 3A**). The cumulative z curve exceeded the benefit boundary with required information size (**Figure 4B**). Detailed comparisons in the DM and non-DM groups were provided in the supplements (**Additional Figure 4D)**.

**4.1.4. AKI**

A total of 74,751 patients with DM were retrieved from 13 studies, of whom 1,622 had AKI during follow-up. The incidence rate of AKI was 2.2%. Compared to placebo users, dapagliflozin users (RR: 0.78, 95% CI: 0.67–0.92) and empagliflozin users (RR: 0.76, 95% CI: 0.63–0.92) were found to have significantly lower risks of AKI (**Figure 3A**). SUCRA ranking is shown in **Additional Figure 2B**. Overall, the cumulative z curve by sequential NMA exceeded the benefit boundary with required information size (**Additional Figure 3B**). Detailed comparisons in the DM and non-DM groups were provided in the supplements (**Additional Figure 1C, 2C, 4E**).

**4.1.5. Other outcomes and side effects**

The incidence rate of side effects, including ketoacidosis, lower limb amputation, urinary tract infection, mycotic genital infection, hypoglycemia, and bone fracture are shown in **Figure 3A**. The details results of comparison between drugs were provided in the Additional files (**Additional Figure 4**).

**4.2. Results by the presence or absence of CKD**

**4.2.1. Kidney-specific composite outcome**

Compared to placebo users, dapagliflozin users (RR: 0.58, 95% CI: 0.48-0.71), empagliflozin users (RR: 0.57, 95% CI: 0.38-0.86), and canagliflozin users (RR: 0.65, 95% CI: 0.52-0.81) were found to have significantly lower risks of the kidney specific composite outcome in the CKD group (**Figure 3B).** The risk of the renal-specific composite outcome was similar between the three SGLT2 inhibitor groups (**Additional Figure 5A**). A total of 6,976 patients without CKD were retrieved from two studies, of whom 179 developed the renal-specific composite outcome during follow-up. Empagliflozin users had a significantly lower risk than placebo users (RR: 0.60, 95% CI: 0.40-0.88) (**Figure 3B**). No significant difference was noted between dapagliflozin and empagliflozin (**Additional Figure 5A).**

**4.2.2. CV death or HHF**

Compared to placebo users, dapagliflozin users (RR: 0.78, 95% CI: 0.71-0.86) and empagliflozin users (RR: 0.84, 95% CI: 0.76-0.93) were found to have significantly lower risks of CV death or HHF **(Figure 3B, Additional Figure 1D)**. No significant difference was noted between dapagliflozin and empagliflozin (**Additional Figure 5B).** The cumulative z curve by TSA exceeded the boundary of benefit with sufficient sample size (**Figure 4A**).

A total of 26,829 patients without CKD were retrieved from five studies, of whom 2,422 developed the composite outcome of CV death or HHF during follow-up. The incidence rate of CV death or HHF was 9.0%. Both dapagliflozin users (RR: 0.84, 95% CI: 0.77-0.92) and empagliflozin users (RR: 0.75, 95% CI: 0.66-0.86) had significantly lower risks of CV death or HHF than placebo users (**Figure 3B, Additional Figure 1E**). No significant difference was noted between dapagliflozin and empagliflozin (**Additional Figure 5B).** The cumulative z curve by TSA exceeded the boundary of benefit with sufficient sample size (**Figure 4A**).

**4.3. *Results by the presence or absence of HF***

**4.3.1. CV death or HHF**

A total of 30,094 patients with HF were retrieved from 10 studies, of whom 2,759 developed the composite outcome of CV death or HHF during follow-up. The incidence rate of CV death or HHF was 9.2%. Sotagliflozin users (RR: 0.73, 95% CI: 0.67-0.80), dapagliflozin users (RR: 0.81, 95% CI: 0.76-0.88), and empagliflozin (RR: 0.79, 95% CI: 0.73-0.86) users had significantly lower risks of CV death or HHF than placebo users (**Figure 3C**). Sotagliflozin users had a lower risk of CV death or HHF than dapagliflozin users (RR: 0.90, 95% CI: 0.80-1.01) (**Figure 2B**). SUCRA ranking demonstrated that sotagliflozin was associated with a lower risk of CV death or HHF (**Additional Figure 2D**). The cumulative z curve exceeded the boundary of benefit and required information size (**Figure 4A**). Detailed comparisons in the HF groups were provided in the supplements (**Additional Figure 5C**).

A total of 32,774 patients without HF were retrieved from four studies, of whom 1,721 developed the composite outcome of CV death or HHF during follow-up. The incidence rate of CV death or HHF was 5.3%. Dapagliflozin (RR: 0.85, 95% CI: 0.72-0.99), canagliflozin (RR: 0.64, 95% CI: 0.51-0.79), and sotagliflozin (RR: 0.72, 95% CI: 0.59-0.89) were associated with lower risks of CV death or HHF **(Figure 3C)**. Among the non-HF patients, those who used canagliflozin had a significantly lower risk of CV death or HHF compared with those who used dapagliflozin (RR: 0.75, 95% CI: 0.58-0.98) **(Figure 2C)**. SUCRA ranking revealed that canagliflozin was associated with a lower risk of CV death or HHF (**Additional** **Figure 2E**). The cumulative z curve by TSA exceeded the boundary of benefit without sufficient sample size (**Figure 4A**). Detailed comparisons in the non-HF groups were provided in the supplements (**Additional Figure 5C**).

**4.3.2. MACEs**

A total of 7,018 patients with MACEs were retrieved from five studies, of whom 1,522 had MACEs during follow-up. The incidence rate of MACE was 21.7%. Among HF patients, sotagliflozin users had a significant lower risk of MACEs than placebo users (RR: 0.76, 95% CI: 0.67-0.85), but dapagliflozin (RR: 1.04, 95% CI: 0.85-1.27), canagliflozin (RR: 0.87, 95% CI: 0.72-1.05) and ertugliflozin users (RR: 1.07, 95% CI: 0.85-1.34) did not **(Figure 3C).** Sotagliflozin users also had a significantly lower risk of MACEs than dapagliflozin users (RR: 0.73, 95% CI: 0.57-0.92) (**Figure 2D**). SUCRA ranking revealed that sotagliflozin was associated with a lower risk of MACEs (**Additional Figure 2F**). Among non-HF patients, only canagliflozin users had significant lower risk of MACE compare with placebo (RR: 0.83, 95% CI: 0.71-0.95) (**Figure 3C**). Detailed comparisons in the HF and non-HF groups were provided in the supplements (**Additional Figure 3C, 5D**).

**5.** **Funnel plot for publication bias by using data of diabetes and non-diabetes.** The minimum number of effect size to calculate Egger’s test is 10.

**Abbreviations:** NA, not applicable.

|  |  |
| --- | --- |
|  |  |
|  |  |
|  |  |
|  |  |
|  |  |

**6. Assessing confidence of evidence according to CINeMA.**

**6.1. Non-diabetes population**

**6.1.1. Death**

eTable. Confidence of evidence assessment for risk of death in non-diabetic population

| Comparison | Number of studies | Within-study bias | Reporting bias | Indirectness | Imprecision | Heterogeneity | Incoherence | Confidence rating |
| --- | --- | --- | --- | --- | --- | --- | --- | --- |
| Placebo:Dapagliflozin | 2 | No concerns | Low risk | No concerns | Major concerns | No concerns | Major concerns | Very low |
| Placebo:Empagliflozin | 1 | No concerns | Low risk | No concerns | Major concerns | No concerns | Major concerns | Very low |
| Dapagliflozin:Empagliflozin | 0 | No concerns | Low risk | No concerns | Major concerns | No concerns | Major concerns | Very low |

**6.1.2. Kidney disease progression**

eTable. Confidence of evidence assessment for risk of kidney disease progression in non-diabetic population

| Comparison | Number of studies | Within-study bias | Reporting bias | Indirectness | Imprecision | Heterogeneity | Incoherence | Confidence rating |
| --- | --- | --- | --- | --- | --- | --- | --- | --- |
| Placebo:Dapagliflozin | 3 | No concerns | Low risk | No concerns | No concerns | Some concerns | Major concerns | Low |
| Placebo:Empagliflozin | 3 | No concerns | Low risk | No concerns | No concerns | Some concerns | Major concerns | Low |
| Dapagliflozin:Empagliflozin | 0 | No concerns | Low risk | No concerns | Major concerns | No concerns | Major concerns | Very low |

**6.1.3. Cardiovascular death or hospitalized heart failure**

eTable. Confidence of evidence assessment for risk of cardiovascular death or hospitalized heart failure in non-diabetic population

| Comparison | Number of studies | Within-study bias | Reporting bias | Indirectness | Imprecision | Heterogeneity | Incoherence | Confidence rating |
| --- | --- | --- | --- | --- | --- | --- | --- | --- |
| Placebo:Dapagliflozin | 3 | No concerns | Low risk | No concerns | No concerns | No concerns | Major concerns | Low |
| Placebo:Empagliflozin | 3 | No concerns | Low risk | No concerns | No concerns | Some concerns | Major concerns | Low |
| Dapagliflozin:Empagliflozin | 0 | No concerns | Low risk | No concerns | Major concerns | No concerns | Major concerns | Very low |

**6.1.4. Cardiovascular death**

eTable. Confidence of evidence assessment for risk of cardiovascular death in non-diabetic population

| Comparison | Number of studies | Within-study bias | Reporting bias | Indirectness | Imprecision | Heterogeneity | Incoherence | Confidence rating |
| --- | --- | --- | --- | --- | --- | --- | --- | --- |
| Placebo:Dapagliflozin | 3 | No concerns | Low risk | No concerns | Some concerns | Some concerns | Major concerns | Very low |
| Placebo:Empagliflozin | 3 | No concerns | Low risk | No concerns | Some concerns | Some concerns | Major concerns | Very low |
| Dapagliflozin:Empagliflozin | 0 | No concerns | Low risk | No concerns | Major concerns | No concerns | Major concerns | Very low |

**6.1.5. Acute kidney injury**

eTable. Confidence of evidence assessment for risk of acute kidney injury in non-diabetic population

| Comparison | Number of studies | Within-study bias | Reporting bias | Indirectness | Imprecision | Heterogeneity | Incoherence | Confidence rating |
| --- | --- | --- | --- | --- | --- | --- | --- | --- |
| Placebo:Dapagliflozin | 3 | No concerns | Low risk | No concerns | No concerns | Some concerns | Major concerns | Low |
| Placebo:Empagliflozin | 3 | No concerns | Low risk | No concerns | No concerns | Some concerns | Major concerns | Low |
| Dapagliflozin:Empagliflozin | 0 | No concerns | Low risk | No concerns | Major concerns | No concerns | Major concerns | Very low |

**6.1.6. Lower limbs amputation**

eTable. Confidence of evidence assessment for risk of lower limbs amputation in non-diabetic population

| Comparison | Number of studies | Within-study bias | Reporting bias | Indirectness | Imprecision | Heterogeneity | Incoherence | Confidence rating |
| --- | --- | --- | --- | --- | --- | --- | --- | --- |
| Placebo:Dapagliflozin | 3 | No concerns | Low risk | No concerns | Major concerns | No concerns | Major concerns | Very low |
| Placebo:Empagliflozin | 3 | No concerns | Low risk | No concerns | Major concerns | No concerns | Major concerns | Very low |
| Dapagliflozin:Empagliflozin | 0 | No concerns | Low risk | No concerns | Major concerns | No concerns | Major concerns | Very low |

**6.2. Diabetes population**

**6.2.1. Death**
eTable. Confidence of evidence assessment for risk of death in diabetic population

| Comparison | Number of studies | Within-study bias | Reporting bias | Indirectness | Imprecision | Heterogeneity | Incoherence | Confidence rating |
| --- | --- | --- | --- | --- | --- | --- | --- | --- |
| Placebo:Dapagliflozin | 4 | No concerns | Low risk | No concerns | No concerns | Some concerns | Major concerns | Low |
| Placebo:Empagliflozin | 2 | No concerns | Low risk | No concerns | No concerns | No concerns | Major concerns | Low |
| Placebo:Canagliflozin | 2 | No concerns | Low risk | No concerns | No concerns | Some concerns | Major concerns | Low |
| Placebo:Sotagliflozin | 2 | No concerns | Low risk | No concerns | Major concerns | No concerns | Major concerns | Very low |
| Placebo:Ertugliflozin | 1 | No concerns | Low risk | No concerns | Major concerns | No concerns | Major concerns | Very low |
| Dapagliflozin:Empagliflozin | 0 | No concerns | Low risk | No concerns | No concerns | Some concerns | Major concerns | Low |
| Dapagliflozin:Canagliflozin | 0 | No concerns | Low risk | No concerns | Major concerns | No concerns | Major concerns | Very low |
| Dapagliflozin:Sotagliflozin | 0 | No concerns | Low risk | No concerns | Major concerns | No concerns | Major concerns | Very low |
| Dapagliflozin:Ertugliflozin | 0 | No concerns | Low risk | No concerns | Major concerns | No concerns | Major concerns | Very low |
| Empagliflozin:Canagliflozin | 0 | No concerns | Low risk | No concerns | Some concerns | Some concerns | Major concerns | Very low |
| Empagliflozin:Sotagliflozin | 0 | No concerns | Low risk | No concerns | No concerns | Some concerns | Major concerns | Low |
| Empagliflozin:Ertugliflozin | 0 | No concerns | Low risk | No concerns | No concerns | Some concerns | Major concerns | Low |
| Canagliflozin:Sotagliflozin | 0 | No concerns | Low risk | No concerns | Major concerns | No concerns | Major concerns | Very low |
| Canagliflozin:Ertugliflozin | 0 | No concerns | Low risk | No concerns | Major concerns | No concerns | Major concerns | Very low |
| Sotagliflozin:Ertugliflozin | 0 | No concerns | Low risk | No concerns | Major concerns | No concerns | Major concerns | Very low |

**6.2.2. Kidney disease progression**

eTable. Confidence of evidence assessment for risk of kidney disease progression in diabetic population

| Comparison | Number of studies | Within-study bias | Reporting bias | Indirectness | Imprecision | Heterogeneity | Incoherence | Confidence rating |
| --- | --- | --- | --- | --- | --- | --- | --- | --- |
| Placebo:Dapagliflozin | 4 | No concerns | Low risk | No concerns | No concerns | No concerns | Major concerns | Low |
| Placebo:Empagliflozin | 4 | No concerns | Low risk | No concerns | No concerns | No concerns | Major concerns | Low |
| Placebo:Canagliflozin | 2 | No concerns | Low risk | No concerns | No concerns | No concerns | Major concerns | Low |
| Placebo:Sotagliflozin | 1 | No concerns | Low risk | No concerns | Some concerns | Some concerns | Major concerns | Very low |
| Placebo:Ertugliflozin | 1 | No concerns | Low risk | No concerns | Major concerns | No concerns | Major concerns | Very low |
| Dapagliflozin:Empagliflozin | 0 | No concerns | Low risk | No concerns | Major concerns | No concerns | Major concerns | Very low |
| Dapagliflozin:Canagliflozin | 0 | No concerns | Low risk | No concerns | Major concerns | No concerns | Major concerns | Very low |
| Dapagliflozin:Sotagliflozin | 0 | No concerns | Low risk | No concerns | Major concerns | No concerns | Major concerns | Very low |
| Dapagliflozin:Ertugliflozin | 0 | No concerns | Low risk | No concerns | Major concerns | No concerns | Major concerns | Very low |
| Empagliflozin:Canagliflozin | 0 | No concerns | Low risk | No concerns | Major concerns | No concerns | Major concerns | Very low |
| Empagliflozin:Sotagliflozin | 0 | No concerns | Low risk | No concerns | Major concerns | No concerns | Major concerns | Very low |
| Empagliflozin:Ertugliflozin | 0 | No concerns | Low risk | No concerns | Major concerns | No concerns | Major concerns | Very low |
| Canagliflozin:Sotagliflozin | 0 | No concerns | Low risk | No concerns | Major concerns | No concerns | Major concerns | Very low |
| Canagliflozin:Ertugliflozin | 0 | No concerns | Low risk | No concerns | Major concerns | No concerns | Major concerns | Very low |
| Sotagliflozin:Ertugliflozin | 0 | No concerns | Low risk | No concerns | Major concerns | No concerns | Major concerns | Very low |

**6.3.3. Cardiovascular death or hospitalized heart failure**

eTable. Confidence of evidence assessment for risk of cardiovascular death or hospitalized heart failure in diabetic population

| Comparison | Number of studies | Within-study bias | Reporting bias | Indirectness | Imprecision | Heterogeneity | Incoherence | Confidence rating |
| --- | --- | --- | --- | --- | --- | --- | --- | --- |
| Placebo:Dapagliflozin | 4 | No concerns | Low risk | No concerns | No concerns | No concerns | Major concerns | Low |
| Placebo:Empagliflozin | 4 | No concerns | Low risk | No concerns | No concerns | No concerns | Major concerns | Low |
| Placebo:Canagliflozin | 2 | No concerns | Low risk | No concerns | No concerns | Some concerns | Major concerns | Low |
| Placebo:Sotagliflozin | 2 | No concerns | Low risk | No concerns | No concerns | No concerns | Major concerns | Low |
| Placebo:Ertugliflozin | 1 | No concerns | Low risk | No concerns | Major concerns | No concerns | Major concerns | Very low |
| Dapagliflozin:Empagliflozin | 0 | No concerns | Low risk | No concerns | Major concerns | No concerns | Major concerns | Very low |
| Dapagliflozin:Canagliflozin | 0 | No concerns | Low risk | No concerns | Major concerns | No concerns | Major concerns | Very low |
| Dapagliflozin:Sotagliflozin | 0 | No concerns | Low risk | No concerns | Major concerns | No concerns | Major concerns | Very low |
| Dapagliflozin:Ertugliflozin | 0 | No concerns | Low risk | No concerns | Major concerns | No concerns | Major concerns | Very low |
| Empagliflozin:Canagliflozin | 0 | No concerns | Low risk | No concerns | Major concerns | No concerns | Major concerns | Very low |
| Empagliflozin:Sotagliflozin | 0 | No concerns | Low risk | No concerns | Major concerns | No concerns | Major concerns | Very low |
| Empagliflozin:Ertugliflozin | 0 | No concerns | Low risk | No concerns | Major concerns | No concerns | Major concerns | Very low |
| Canagliflozin:Sotagliflozin | 0 | No concerns | Low risk | No concerns | Major concerns | No concerns | Major concerns | Very low |
| Canagliflozin:Ertugliflozin | 0 | No concerns | Low risk | No concerns | Major concerns | No concerns | Major concerns | Very low |
| Sotagliflozin:Ertugliflozin | 0 | No concerns | Low risk | No concerns | Some concerns | Some concerns | Major concerns | Very low |

**6.3.4. Cardiovascular death**

eTable. Confidence of evidence assessment for risk of cardiovascular death in diabetic population

| Comparison | Number of studies | Within-study bias | Reporting bias | Indirectness | Imprecision | Heterogeneity | Incoherence | Confidence rating |
| --- | --- | --- | --- | --- | --- | --- | --- | --- |
| Placebo:Dapagliflozin | 4 | No concerns | Low risk | No concerns | Some concerns | Some concerns | Major concerns | Very low |
| Placebo:Empagliflozin | 4 | No concerns | Low risk | No concerns | No concerns | Major concerns | Major concerns | Very low |
| Placebo:Canagliflozin | 2 | No concerns | Low risk | No concerns | Major concerns | No concerns | Major concerns | Very low |
| Placebo:Sotagliflozin | 2 | No concerns | Low risk | No concerns | Major concerns | No concerns | Major concerns | Very low |
| Placebo:Ertugliflozin | 1 | No concerns | Low risk | No concerns | Major concerns | No concerns | Major concerns | Very low |
| Dapagliflozin:Empagliflozin | 0 | No concerns | Low risk | No concerns | Major concerns | No concerns | Major concerns | Very low |
| Dapagliflozin:Canagliflozin | 0 | No concerns | Low risk | No concerns | Major concerns | No concerns | Major concerns | Very low |
| Dapagliflozin:Sotagliflozin | 0 | No concerns | Low risk | No concerns | Major concerns | No concerns | Major concerns | Very low |
| Dapagliflozin:Ertugliflozin | 0 | No concerns | Low risk | No concerns | Major concerns | No concerns | Major concerns | Very low |
| Empagliflozin:Canagliflozin | 0 | No concerns | Low risk | No concerns | Major concerns | No concerns | Major concerns | Very low |
| Empagliflozin:Sotagliflozin | 0 | No concerns | Low risk | No concerns | Major concerns | No concerns | Major concerns | Very low |
| Empagliflozin:Ertugliflozin | 0 | No concerns | Low risk | No concerns | Major concerns | No concerns | Major concerns | Very low |
| Canagliflozin:Sotagliflozin | 0 | No concerns | Low risk | No concerns | Major concerns | No concerns | Major concerns | Very low |
| Canagliflozin:Ertugliflozin | 0 | No concerns | Low risk | No concerns | Major concerns | No concerns | Major concerns | Very low |
| Sotagliflozin:Ertugliflozin | 0 | No concerns | Low risk | No concerns | Major concerns | No concerns | Major concerns | Very low |

**6.3.5. Acute kidney injury**

eTable. Confidence of evidence assessment for risk of acute kidney injury in diabetic population

| Comparison | Number of studies | Within-study bias | Reporting bias | Indirectness | Imprecision | Heterogeneity | Incoherence | Confidence rating |
| --- | --- | --- | --- | --- | --- | --- | --- | --- |
| Placebo:Dapagliflozin | 4 | No concerns | Low risk | No concerns | No concerns | No concerns | Major concerns | Low |
| Placebo:Empagliflozin | 4 | No concerns | Low risk | No concerns | No concerns | No concerns | Major concerns | Low |
| Placebo:Canagliflozin | 2 | No concerns | Low risk | No concerns | Some concerns | Some concerns | Major concerns | Very low |
| Placebo:Sotagliflozin | 2 | No concerns | Low risk | No concerns | Major concerns | No concerns | Major concerns | Very low |
| Placebo:Ertugliflozin | 1 | No concerns | Low risk | No concerns | Major concerns | No concerns | Major concerns | Very low |
| Dapagliflozin:Empagliflozin | 0 | No concerns | Low risk | No concerns | Major concerns | No concerns | Major concerns | Very low |
| Dapagliflozin:Canagliflozin | 0 | No concerns | Low risk | No concerns | Major concerns | No concerns | Major concerns | Very low |
| Dapagliflozin:Sotagliflozin | 0 | No concerns | Low risk | No concerns | Some concerns | No concerns | Major concerns | Low |
| Dapagliflozin:Ertugliflozin | 0 | No concerns | Low risk | No concerns | Major concerns | No concerns | Major concerns | Very low |
| Empagliflozin:Canagliflozin | 0 | No concerns | Low risk | No concerns | Major concerns | No concerns | Major concerns | Very low |
| Empagliflozin:Sotagliflozin | 0 | No concerns | Low risk | No concerns | Some concerns | No concerns | Major concerns | Low |
| Empagliflozin:Ertugliflozin | 0 | No concerns | Low risk | No concerns | Major concerns | No concerns | Major concerns | Very low |
| Canagliflozin:Sotagliflozin | 0 | No concerns | Low risk | No concerns | Major concerns | No concerns | Major concerns | Very low |
| Canagliflozin:Ertugliflozin | 0 | No concerns | Low risk | No concerns | Major concerns | No concerns | Major concerns | Very low |
| Sotagliflozin:Ertugliflozin | 0 | No concerns | Low risk | No concerns | Major concerns | No concerns | Major concerns | Very low |

**6.3.6. Ketoacidosis**

eTable. Confidence of evidence assessment for risk of ketoacidosis in diabetic population

| Comparison | Number of studies | Within-study bias | Reporting bias | Indirectness | Imprecision | Heterogeneity | Incoherence | Confidence rating |
| --- | --- | --- | --- | --- | --- | --- | --- | --- |
| Placebo:Dapagliflozin | 4 | No concerns | Low risk | No concerns | Major concerns | No concerns | Major concerns | Very low |
| Placebo:Empagliflozin | 4 | No concerns | Low risk | No concerns | Major concerns | No concerns | Major concerns | Very low |
| Placebo:Canagliflozin | 2 | No concerns | Low risk | No concerns | Some concerns | Some concerns | Major concerns | Very low |
| Placebo:Sotagliflozin | 2 | No concerns | Low risk | No concerns | Major concerns | No concerns | Major concerns | Very low |
| Placebo:Ertugliflozin | 1 | No concerns | Low risk | No concerns | Major concerns | No concerns | Major concerns | Very low |
| Dapagliflozin:Empagliflozin | 0 | No concerns | Low risk | No concerns | Major concerns | No concerns | Major concerns | Very low |
| Dapagliflozin:Canagliflozin | 0 | No concerns | Low risk | No concerns | Major concerns | No concerns | Major concerns | Very low |
| Dapagliflozin:Sotagliflozin | 0 | No concerns | Low risk | No concerns | Major concerns | No concerns | Major concerns | Very low |
| Dapagliflozin:Ertugliflozin | 0 | No concerns | Low risk | No concerns | Major concerns | No concerns | Major concerns | Very low |
| Empagliflozin:Canagliflozin | 0 | No concerns | Low risk | No concerns | Major concerns | No concerns | Major concerns | Very low |
| Empagliflozin:Sotagliflozin | 0 | No concerns | Low risk | No concerns | Major concerns | No concerns | Major concerns | Very low |
| Empagliflozin:Ertugliflozin | 0 | No concerns | Low risk | No concerns | Major concerns | No concerns | Major concerns | Very low |
| Canagliflozin:Sotagliflozin | 0 | No concerns | Low risk | No concerns | Major concerns | No concerns | Major concerns | Very low |
| Canagliflozin:Ertugliflozin | 0 | No concerns | Low risk | No concerns | Major concerns | No concerns | Major concerns | Very low |
| Sotagliflozin:Ertugliflozin | 0 | No concerns | Low risk | No concerns | Major concerns | No concerns | Major concerns | Very low |

**6.3.7. Lower limbs amputation**

eTable. Confidence of evidence assessment for risk of lower limbs amputation in diabetic population

| Comparison | Number of studies | Within-study bias | Reporting bias | Indirectness | Imprecision | Heterogeneity | Incoherence | Confidence rating |
| --- | --- | --- | --- | --- | --- | --- | --- | --- |
| Placebo:Dapagliflozin | 4 | No concerns | Low risk | No concerns | No concerns | No concerns | Major concerns | Low |
| Placebo:Empagliflozin | 4 | No concerns | Low risk | No concerns | No concerns | No concerns | Major concerns | Low |
| Placebo:Canagliflozin | 2 | No concerns | Low risk | No concerns | No concerns | No concerns | Major concerns | Low |
| Placebo:Sotagliflozin | 2 | No concerns | Low risk | No concerns | No concerns | No concerns | Major concerns | Low |
| Placebo:Ertugliflozin | 1 | No concerns | Low risk | No concerns | No concerns | No concerns | Major concerns | Low |
| Dapagliflozin:Empagliflozin | 0 | No concerns | Low risk | No concerns | No concerns | No concerns | Major concerns | Low |
| Dapagliflozin:Canagliflozin | 0 | No concerns | Low risk | No concerns | No concerns | No concerns | Major concerns | Low |
| Dapagliflozin:Sotagliflozin | 0 | No concerns | Low risk | No concerns | No concerns | No concerns | Major concerns | Low |
| Dapagliflozin:Ertugliflozin | 0 | No concerns | Low risk | No concerns | No concerns | No concerns | Major concerns | Low |
| Empagliflozin:Canagliflozin | 0 | No concerns | Low risk | No concerns | No concerns | No concerns | Major concerns | Low |
| Empagliflozin:Sotagliflozin | 0 | No concerns | Low risk | No concerns | No concerns | No concerns | Major concerns | Low |
| Empagliflozin:Ertugliflozin | 0 | No concerns | Low risk | No concerns | No concerns | No concerns | Major concerns | Low |
| Canagliflozin:Sotagliflozin | 0 | No concerns | Low risk | No concerns | No concerns | No concerns | Major concerns | Low |
| Canagliflozin:Ertugliflozin | 0 | No concerns | Low risk | No concerns | No concerns | No concerns | Major concerns | Low |
| Sotagliflozin:Ertugliflozin | 0 | No concerns | Low risk | No concerns | No concerns | No concerns | Major concerns | Low |

**6.3.8. Urinary tract infection**

eTable. Confidence of evidence assessment for risk of urinary tract infection in diabetic population

| Comparison | Number of studies | Within-study bias | Reporting bias | Indirectness | Imprecision | Heterogeneity | Incoherence | Confidence rating |
| --- | --- | --- | --- | --- | --- | --- | --- | --- |
| Placebo:Dapagliflozin | 1 | No concerns | Low risk | No concerns | Major concerns | No concerns | Major concerns | Very low |
| Placebo:Empagliflozin | 2 | No concerns | Low risk | No concerns | Major concerns | No concerns | Major concerns | Very low |
| Placebo:Canagliflozin | 2 | No concerns | Low risk | No concerns | Some concerns | Some concerns | Major concerns | Very low |
| Placebo:Sotagliflozin | 2 | No concerns | Low risk | No concerns | Some concerns | Some concerns | Major concerns | Very low |
| Placebo:Ertugliflozin | 1 | No concerns | Low risk | No concerns | No concerns | Major concerns | Major concerns | Very low |
| Dapagliflozin:Empagliflozin | 0 | No concerns | Low risk | No concerns | Major concerns | No concerns | Major concerns | Very low |
| Dapagliflozin:Canagliflozin | 0 | No concerns | Low risk | No concerns | Major concerns | No concerns | Major concerns | Very low |
| Dapagliflozin:Sotagliflozin | 0 | No concerns | Low risk | No concerns | Major concerns | No concerns | Major concerns | Very low |
| Dapagliflozin:Ertugliflozin | 0 | No concerns | Low risk | No concerns | Some concerns | Some concerns | Major concerns | Very low |
| Empagliflozin:Canagliflozin | 0 | No concerns | Low risk | No concerns | Major concerns | No concerns | Major concerns | Very low |
| Empagliflozin:Sotagliflozin | 0 | No concerns | Low risk | No concerns | Major concerns | No concerns | Major concerns | Very low |
| Empagliflozin:Ertugliflozin | 0 | No concerns | Low risk | No concerns | Major concerns | No concerns | Major concerns | Very low |
| Canagliflozin:Sotagliflozin | 0 | No concerns | Low risk | No concerns | Major concerns | No concerns | Major concerns | Very low |
| Canagliflozin:Ertugliflozin | 0 | No concerns | Low risk | No concerns | Some concerns | Some concerns | Major concerns | Very low |
| Sotagliflozin:Ertugliflozin | 0 | No concerns | Low risk | No concerns | Some concerns | Some concerns | Major concerns | Very low |

**6.3.9. Mycotic genital infections**

eTable. Confidence of evidence assessment for risk of mycotic genital infections in diabetic population

| Comparison | Number of studies | Within-study bias | Reporting bias | Indirectness | Imprecision | Heterogeneity | Incoherence | Confidence rating |
| --- | --- | --- | --- | --- | --- | --- | --- | --- |
| Placebo:Dapagliflozin | 1 | No concerns | Low risk | No concerns | Major concerns | No concerns | Major concerns | Very low |
| Placebo:Empagliflozin | 2 | No concerns | Low risk | No concerns | No concerns | No concerns | Major concerns | Low |
| Placebo:Canagliflozin | 2 | No concerns | Low risk | No concerns | No concerns | No concerns | Major concerns | Low |
| Placebo:Sotagliflozin | 2 | No concerns | Low risk | No concerns | No concerns | No concerns | Major concerns | Low |
| Placebo:Ertugliflozin | 1 | No concerns | Low risk | No concerns | No concerns | No concerns | Major concerns | Low |
| Dapagliflozin:Empagliflozin | 0 | No concerns | Low risk | No concerns | Major concerns | No concerns | Major concerns | Very low |
| Dapagliflozin:Canagliflozin | 0 | No concerns | Low risk | No concerns | Major concerns | No concerns | Major concerns | Very low |
| Dapagliflozin:Sotagliflozin | 0 | No concerns | Low risk | No concerns | Major concerns | No concerns | Major concerns | Very low |
| Dapagliflozin:Ertugliflozin | 0 | No concerns | Low risk | No concerns | Major concerns | No concerns | Major concerns | Very low |
| Empagliflozin:Canagliflozin | 0 | No concerns | Low risk | No concerns | Major concerns | No concerns | Major concerns | Very low |
| Empagliflozin:Sotagliflozin | 0 | No concerns | Low risk | No concerns | Major concerns | No concerns | Major concerns | Very low |
| Empagliflozin:Ertugliflozin | 0 | No concerns | Low risk | No concerns | Major concerns | No concerns | Major concerns | Very low |
| Canagliflozin:Sotagliflozin | 0 | No concerns | Low risk | No concerns | Some concerns | Some concerns | Major concerns | Very low |
| Canagliflozin:Ertugliflozin | 0 | No concerns | Low risk | No concerns | Major concerns | No concerns | Major concerns | Very low |
| Sotagliflozin:Ertugliflozin | 0 | No concerns | Low risk | No concerns | Major concerns | No concerns | Major concerns | Very low |

**6.3.10. Hypoglycemia**

eTable. Confidence of evidence assessment for risk of hypoglycemia in diabetic population

| Comparison | Number of studies | Within-study bias | Reporting bias | Indirectness | Imprecision | Heterogeneity | Incoherence | Confidence rating |
| --- | --- | --- | --- | --- | --- | --- | --- | --- |
| Placebo:Dapagliflozin | 2 | No concerns | Low risk | No concerns | Some concerns | Some concerns | Major concerns | Very low |
| Placebo:Empagliflozin | 3 | No concerns | Low risk | No concerns | Major concerns | No concerns | Major concerns | Very low |
| Placebo:Canagliflozin | 2 | No concerns | Low risk | No concerns | Some concerns | Some concerns | Major concerns | Very low |
| Placebo:Sotagliflozin | 2 | No concerns | Low risk | No concerns | Major concerns | No concerns | Major concerns | Very low |
| Placebo:Ertugliflozin | 1 | No concerns | Low risk | No concerns | Some concerns | Some concerns | Major concerns | Very low |
| Dapagliflozin:Empagliflozin | 0 | No concerns | Low risk | No concerns | Some concerns | Some concerns | Major concerns | Very low |
| Dapagliflozin:Canagliflozin | 0 | No concerns | Low risk | No concerns | Some concerns | Some concerns | Major concerns | Very low |
| Dapagliflozin:Sotagliflozin | 0 | No concerns | Low risk | No concerns | Major concerns | No concerns | Major concerns | Very low |
| Dapagliflozin:Ertugliflozin | 0 | No concerns | Low risk | No concerns | Some concerns | Some concerns | Major concerns | Very low |
| Empagliflozin:Canagliflozin | 0 | No concerns | Low risk | No concerns | Major concerns | No concerns | Major concerns | Very low |
| Empagliflozin:Sotagliflozin | 0 | No concerns | Low risk | No concerns | Major concerns | No concerns | Major concerns | Very low |
| Empagliflozin:Ertugliflozin | 0 | No concerns | Low risk | No concerns | Major concerns | No concerns | Major concerns | Very low |
| Canagliflozin:Sotagliflozin | 0 | No concerns | Low risk | No concerns | Major concerns | No concerns | Major concerns | Very low |
| Canagliflozin:Ertugliflozin | 0 | No concerns | Low risk | No concerns | Major concerns | No concerns | Major concerns | Very low |
| Sotagliflozin:Ertugliflozin | 0 | No concerns | Low risk | No concerns | Major concerns | No concerns | Major concerns | Very low |

**6.3.11. Bone fracture**

eTable. Confidence of evidence assessment for risk of bone fracture in diabetic population

| Comparison | Number of studies | Within-study bias | Reporting bias | Indirectness | Imprecision | Heterogeneity | Incoherence | Confidence rating |
| --- | --- | --- | --- | --- | --- | --- | --- | --- |
| Placebo:Dapagliflozin | 2 | No concerns | Low risk | No concerns | No concerns | No concerns | Major concerns | Low |
| Placebo:Empagliflozin | 2 | No concerns | Low risk | No concerns | No concerns | No concerns | Major concerns | Low |
| Placebo:Canagliflozin | 2 | No concerns | Low risk | No concerns | No concerns | No concerns | Major concerns | Low |
| Placebo:Sotagliflozin | 2 | No concerns | Low risk | No concerns | No concerns | No concerns | Major concerns | Low |
| Placebo:Ertugliflozin | 1 | No concerns | Low risk | No concerns | No concerns | No concerns | Major concerns | Low |
| Dapagliflozin:Empagliflozin | 0 | No concerns | Low risk | No concerns | No concerns | No concerns | Major concerns | Low |
| Dapagliflozin:Canagliflozin | 0 | No concerns | Low risk | No concerns | No concerns | No concerns | Major concerns | Low |
| Dapagliflozin:Sotagliflozin | 0 | No concerns | Low risk | No concerns | No concerns | No concerns | Major concerns | Low |
| Dapagliflozin:Ertugliflozin | 0 | No concerns | Low risk | No concerns | No concerns | No concerns | Major concerns | Low |
| Empagliflozin:Canagliflozin | 0 | No concerns | Low risk | No concerns | No concerns | No concerns | Major concerns | Low |
| Empagliflozin:Sotagliflozin | 0 | No concerns | Low risk | No concerns | No concerns | No concerns | Major concerns | Low |
| Empagliflozin:Ertugliflozin | 0 | No concerns | Low risk | No concerns | No concerns | No concerns | Major concerns | Low |
| Canagliflozin:Sotagliflozin | 0 | No concerns | Low risk | No concerns | No concerns | No concerns | Major concerns | Low |
| Canagliflozin:Ertugliflozin | 0 | No concerns | Low risk | No concerns | No concerns | No concerns | Major concerns | Low |
| Sotagliflozin:Ertugliflozin | 0 | No concerns | Low risk | No concerns | No concerns | No concerns | Major concerns | Low |

**6.3. Chronic kidney disease population**

**6.3.1. Renal-specific composite outcomes**

eTable. Confidence of evidence assessment for risk of renal-specific composite outcomes in chronic kidney disease population

| Comparison | Number of studies | Within-study bias | Reporting bias | Indirectness | Imprecision | Heterogeneity | Incoherence | Confidence rating |
| --- | --- | --- | --- | --- | --- | --- | --- | --- |
| Placebo:Dapagliflozin | 1 | No concerns | Low risk | No concerns | No concerns | Major concerns | Major concerns | Very low |
| Placebo:Empagliflozin | 1 | No concerns | Low risk | No concerns | No concerns | Major concerns | Major concerns | Very low |
| Placebo:Canagliflozin | 1 | No concerns | Low risk | No concerns | No concerns | Major concerns | Major concerns | Very low |
| Dapagliflozin:Empagliflozin | 0 | No concerns | Low risk | No concerns | Major concerns | No concerns | Major concerns | Very low |
| Dapagliflozin:Canagliflozin | 0 | No concerns | Low risk | No concerns | Major concerns | No concerns | Major concerns | Very low |
| Empagliflozin:Canagliflozin | 0 | No concerns | Low risk | No concerns | Major concerns | No concerns | Major concerns | Very low |

**6.3.2. Cardiovascular death or hospitalized heart failure**

eTable. Confidence of evidence assessment for risk of cardiovascular death or hospitalized heart failure in chronic kidney disease population

| Comparison | Number of studies | Within-study bias | Reporting bias | Indirectness | Imprecision | Heterogeneity | Incoherence | Confidence rating |
| --- | --- | --- | --- | --- | --- | --- | --- | --- |
| Placebo:Dapagliflozin | 4 | No concerns | Low risk | No concerns | No concerns | No concerns | Major concerns | Low |
| Placebo:Empagliflozin | 3 | No concerns | Low risk | No concerns | No concerns | No concerns | Major concerns | Low |
| Dapagliflozin:Empagliflozin | 0 | No concerns | Low risk | No concerns | No concerns | No concerns | Major concerns | Low |

**6.4. Non-chronic kidney disease population**

**6.4.1. Renal-specific composite outcomes**

eTable. Confidence of evidence assessment for risk of renal-specific composite outcomes in non-chronic kidney disease population

| Comparison | Number of studies | Within-study bias | Reporting bias | Indirectness | Imprecision | Heterogeneity | Incoherence | Confidence rating |
| --- | --- | --- | --- | --- | --- | --- | --- | --- |
| Placebo:Empagliflozin | 1 | No concerns | Low risk | No concerns | No concerns | Major concerns | Major concerns | Very low |
| Placebo:Canagliflozin | 1 | No concerns | Low risk | No concerns | Major concerns | No concerns | Major concerns | Very low |
| Empagliflozin:Canagliflozin | 0 | No concerns | Low risk | No concerns | Major concerns | No concerns | Major concerns | Very low |

**6.4.2. Cardiovascular death or hospitalized heart failure**

eTable. Confidence of evidence assessment for risk of cardiovascular disease or hospitalized heart failure in non-chronic kidney disease population

| Comparison | Number of studies | Within-study bias | Reporting bias | Indirectness | Imprecision | Heterogeneity | Incoherence | Confidence rating |
| --- | --- | --- | --- | --- | --- | --- | --- | --- |
| Placebo:Dapagliflozin | 3 | No concerns | Low risk | No concerns | No concerns | Some concerns | Major concerns | Low |
| Placebo:Empagliflozin | 2 | No concerns | Low risk | No concerns | No concerns | Some concerns | Major concerns | Low |
| Dapagliflozin:Empagliflozin | 0 | No concerns | Low risk | No concerns | Some concerns | Some concerns | Major concerns | Very low |

**6.5 Heart failure population**

**6.5.1. Major adverse cardiovascular events**

eTable. Confidence of evidence assessment for major adverse cardiovascular events in heart failure population

| Comparison | Number of studies | Within-study bias | Reporting bias | Indirectness | Imprecision | Heterogeneity | Incoherence | Confidence rating |
| --- | --- | --- | --- | --- | --- | --- | --- | --- |
| Placebo:Dapagliflozin | 1 | No concerns | Low risk | No concerns | Major concerns | No concerns | Major concerns | Very low |
| Placebo:Canagliflozin | 2 | No concerns | Low risk | No concerns | Some concerns | Some concerns | Major concerns | Very low |
| Placebo:Sotagliflozin | 1 | No concerns | Low risk | No concerns | No concerns | Major concerns | Major concerns | Very low |
| Placebo:Ertugliflozin | 1 | No concerns | Low risk | No concerns | Major concerns | No concerns | Major concerns | Very low |
| Dapagliflozin:Canagliflozin | 0 | No concerns | Low risk | No concerns | Some concerns | Some concerns | Major concerns | Very low |
| Dapagliflozin:Sotagliflozin | 0 | No concerns | Low risk | No concerns | No concerns | Major concerns | Major concerns | Very low |
| Dapagliflozin:Ertugliflozin | 0 | No concerns | Low risk | No concerns | Major concerns | No concerns | Major concerns | Very low |
| Canagliflozin:Sotagliflozin | 0 | No concerns | Low risk | No concerns | Some concerns | Some concerns | Major concerns | Very low |
| Canagliflozin:Ertugliflozin | 0 | No concerns | Low risk | No concerns | Some concerns | Some concerns | Major concerns | Very low |
| Sotagliflozin:Ertugliflozin | 0 | No concerns | Low risk | No concerns | No concerns | Major concerns | Major concerns | Very low |

**6.5.2. Cardiovascular death or hospitalized heart failure**

eTable. Confidence of evidence assessment for cardiovascular death or hospitalized heart failure in heart failure population

| Comparison | Number of studies | Within-study bias | Reporting bias | Indirectness | Imprecision | Heterogeneity | Incoherence | Confidence rating |
| --- | --- | --- | --- | --- | --- | --- | --- | --- |
| Placebo:Dapagliflozin | 3 | No concerns | Low risk | No concerns | No concerns | No concerns | Major concerns | Low |
| Placebo:Empagliflozin | 3 | No concerns | Low risk | No concerns | No concerns | No concerns | Major concerns | Low |
| Placebo:Canagliflozin | 1 | No concerns | Low risk | No concerns | Major concerns | No concerns | Major concerns | Very low |
| Placebo:Sotagliflozin | 2 | No concerns | Low risk | No concerns | No concerns | No concerns | Major concerns | Low |
| Placebo:Ertugliflozin | 1 | No concerns | Low risk | No concerns | Some concerns | Some concerns | Major concerns | Very low |
| Dapagliflozin:Empagliflozin | 0 | No concerns | Low risk | No concerns | Some concerns | Some concerns | Major concerns | Very low |
| Dapagliflozin:Canagliflozin | 0 | No concerns | Low risk | No concerns | Major concerns | No concerns | Major concerns | Very low |
| Dapagliflozin:Sotagliflozin | 0 | No concerns | Low risk | No concerns | Some concerns | No concerns | Major concerns | Low |
| Dapagliflozin:Ertugliflozin | 0 | No concerns | Low risk | No concerns | Major concerns | No concerns | Major concerns | Very low |
| Empagliflozin:Canagliflozin | 0 | No concerns | Low risk | No concerns | Major concerns | No concerns | Major concerns | Very low |
| Empagliflozin:Sotagliflozin | 0 | No concerns | Low risk | No concerns | Some concerns | No concerns | Major concerns | Low |
| Empagliflozin:Ertugliflozin | 0 | No concerns | Low risk | No concerns | Major concerns | No concerns | Major concerns | Very low |
| Canagliflozin:Sotagliflozin | 0 | No concerns | Low risk | No concerns | Major concerns | No concerns | Major concerns | Very low |
| Canagliflozin:Ertugliflozin | 0 | No concerns | Low risk | No concerns | Major concerns | No concerns | Major concerns | Very low |
| Sotagliflozin:Ertugliflozin | 0 | No concerns | Low risk | No concerns | Some concerns | Some concerns | Major concerns | Very low |

**6.6 Non-heart failure population**

**6.6.1. Major adverse cardiovascular events**

eTable. Confidence of evidence assessment for major adverse cardiovascular events in non-heart failure population

| Comparison | Number of studies | Within-study bias | Reporting bias | Indirectness | Imprecision | Heterogeneity | Incoherence | Confidence rating |
| --- | --- | --- | --- | --- | --- | --- | --- | --- |
| Placebo:Dapagliflozin | 1 | No concerns | Low risk | No concerns | Some concerns | Some concerns | Major concerns | Very low |
| Placebo:Canagliflozin | 2 | No concerns | Low risk | No concerns | No concerns | Major concerns | Major concerns | Very low |
| Placebo:Ertugliflozin | 1 | No concerns | Low risk | No concerns | Major concerns | No concerns | Major concerns | Very low |
| Dapagliflozin:Canagliflozin | 0 | No concerns | Low risk | No concerns | Major concerns | No concerns | Major concerns | Very low |
| Dapagliflozin:Ertugliflozin | 0 | No concerns | Low risk | No concerns | Major concerns | No concerns | Major concerns | Very low |
| Canagliflozin:Ertugliflozin | 0 | No concerns | Low risk | No concerns | Some concerns | Some concerns | Major concerns | Very low |

**6.6.2. Cardiovascular death or hospitalized heart failure**

eTable. Confidence of evidence assessment for cardiovascular death or hospitalized heart failure in non-heart failure population

| Comparison | Number of studies | Within-study bias | Reporting bias | Indirectness | Imprecision | Heterogeneity | Incoherence | Confidence rating |
| --- | --- | --- | --- | --- | --- | --- | --- | --- |
| Placebo:Dapagliflozin | 1 | No concerns | Low risk | No concerns | No concerns | Major concerns | Major concerns | Very low |
| Placebo:Canagliflozin | 1 | No concerns | Low risk | No concerns | No concerns | Major concerns | Major concerns | Very low |
| Placebo:Sotagliflozin | 1 | No concerns | Low risk | No concerns | No concerns | Major concerns | Major concerns | Very low |
| Placebo:Ertugliflozin | 1 | No concerns | Low risk | No concerns | Major concerns | No concerns | Major concerns | Very low |
| Dapagliflozin:Canagliflozin | 0 | No concerns | Low risk | No concerns | No concerns | Major concerns | Major concerns | Very low |
| Dapagliflozin:Sotagliflozin | 0 | No concerns | Low risk | No concerns | Some concerns | Some concerns | Major concerns | Very low |
| Dapagliflozin:Ertugliflozin | 0 | No concerns | Low risk | No concerns | Major concerns | No concerns | Major concerns | Very low |
| Canagliflozin:Sotagliflozin | 0 | No concerns | Low risk | No concerns | Major concerns | No concerns | Major concerns | Very low |
| Canagliflozin:Ertugliflozin | 0 | No concerns | Low risk | No concerns | No concerns | Major concerns | Major concerns | Very low |
| Sotagliflozin:Ertugliflozin | 0 | No concerns | Low risk | No concerns | Some concerns | Some concerns | Major concerns | Very low |

**7. PROSPERO protocol registration**

**The Impact of Sodium-Glucose Co-transporter-2 Inhibitors on Mortality and Cardiovascular**

**Outcomes Among non-DM Adults: a systematic review and meta-analysis**

Review question

P: non-DM patients, I: SGLT2 inhibitor, C:placebo, O:mortality and CV outcomes

Searches

PubMed, Embase, MEDLINE, Cochrane

Types of study to be included

RCT

Condition or domain being studied

SGLT2 inhibitor, mortality and CV outcomes

Participants/population

The inclusion criteria as follows: 1. human studies aged more than 18 years or older were randomized into control or

experimental study group 2. non-DM patients, 3.Comparison of the relevant outcomes between two groups, including

mortality and CV outcomes. The papers were not RCT, or those were DM patients were excluded.

Intervention(s), exposure(s)

inclusion criteria:non-DM patients with SGLT2 inhibitor; exclusion criteria: DM patients

Comparator(s)/control

non-DM patients without using SGLT2 inhibitor

Context

non-DM patients

Main outcome(s)

the risk of mortality

Measures of effect

Odds ratios

Additional outcome(s)

CV outcomes including CV death and Heart failure hospitalization

Measures of effect

Odds ratio

Data extraction (selection and coding)

The following data were extracted from the full-text articles: the first author name, year of publication, sample size,

study design, patient inclusion criteria, patient demographics, and clinical outcomes.

Risk of bias (quality) assessment

The Cochrane risk of bias tool was used for quality assessment of RCTs. The following domains were assessed: random

sequence generation, allocation concealment, blinding of participants and personnel, blinding of outcome assessment, incomplete outcome data, selective reporting and other bias

Strategy for data synthesis

Between-trial heterogeneity was determined by using I² tests, and values > 50% were regarded as considerable

heterogeneity.

Analysis of subgroups or subsets

We performed subgroup analysis for primary outcome about IgA nephropathy or study quality.

Contact details for further information

Jui-Yi Chen

kwuilus0101@gmail.com

Organisational affiliation of the review

Chi-Mei Medical Center

Review team members and their organisational affiliations

Mr Jui-Yi Chen. Chi-Mei Medical Center

Vin-Cent Wu. Department of Internal Medicine, National Taiwan University Hospital

Type and method of review

Intervention, Meta-analysis, Systematic review

Anticipated or actual start date

15 September 2022

Anticipated completion date

06 November 2022

Funding sources/sponsors

nil

Grant number(s) State the funder, grant or award number and the date of award

nil

Language

English

Country

Taiwan

Stage of review

Review Ongoing

Subject index terms status

Subject indexing assigned by CRD

Subject index terms

Glucose; Humans; Hypoglycemic Agents; Sodium; Sodium-Glucose Transporter 2; Sodium-Glucose Transporter 2

Inhibitors

Date of registration in PROSPERO

06 October 2022

Date of first submission

25 September 2022

Stage of review at time of this submission

| **Stage** | **Started** | **Completed** |
| --- | --- | --- |
| Preliminary searches | **Yes** | **No** |
| Piloting of the study selection process | **Yes** | **No** |
| Formal screening of search results against eligibility criteria | **Yes** | **No** |
| Data extraction | **No** | **No** |
| Risk of bias (quality) assessment | **No** | **No** |
| Data analysis | **No** | **No** |

**8. Summary of the feature in each study**

In our study, a total 14 RCTs with 75,334 participants with complete data and outcomes of interest, were enrolled for the final meta‐analysis.

**For analysis of the clinical effect of SGLT-2 inhibitors for patients with type 2 diabetes at high ASVD risk, 4 studies were included.**

**DECLARE-TIMI 58 (2009)** randomly assigned 17160 patients (10.0% HF, 7.4% CKD) to receive dapagliflozin or placebo. Compared with placebo users, the HR for the MACE, composite of CV outcomes, cardiovascular death of dapagliflozin users were 0.93 (95% CI: 0.84 to 1.03), 0.83 (95% CI: 0.73 to 0.95), and 0.98 (95% CI: 0.82 to 1.17), respectively. Dapagliflozin users also had significantly lower risk of kidney event (HR: 0.76, 95% CI: 0.67 to 0.87) but did not have significantly lower risk of death from any causes (HR: 0.93, 95% CI: 0.82 to 1.04) than placebo users.

**EMPA-REG OUTCOME (2015) r**andomly assigned 7020 patients (25.9% CKD) to receive empagliflozin (5 mg or 15 mg once daily) or placebo. Empagliflozin users had a significantly lower risk of MACE (HR: 0.86, 95% CI: 0.74 to 0.99) than placebo users. Compared with placebo users, empagliflozin users had a 38%, 35%, and 32% relative risk reduction in death from cardiovascular causes, hospitalization for heart failure, and death from any cause, respectively.

**CANVAS Program (2017) r**andomly assigned 17160 patients (14.5% HF) to receive canagliflozin or placebo. Compared with placebo users, the HR for the MACE, progression of albuminuria, the composite of kidney outcomes of canagliflozin users were 0.86 (95% CI: 0.75 to 0.97), 0.73 (95% CI: 0.67 to 0.79), and 0.60 (95% CI: 0.47 to 0.77), respectively. However, canagliflozin users also had an increased risk of amputation 1.97 (95% CI: 1.41 to 1.75) than placebo users.

**VERTIS CV (2020)** randomly assigned 8246 patients (23.8% HF, 21.9% CKD) to receive ertugliflozin (5 mg or 15 mg once daily) or placebo. Compared with placebo users, the HR for the MACE, composite of CV outcomes, and composite of kidney outcomes of ertugliflozin users were 0.97 (95% CI: 0.85 to 1.11), 0.88 (95% CI: 0.75 to 1.03), and 0.81 (95% CI: 0.63 to 1.04), respectively.

**For analysis of the clinical effect of SGLT-2 inhibitors for patients with heart failure, 5 studies were included.**

**DAPA-HF (2019)** randomized 4,744 HF patients (41.8% diabetic, 40.7% CKD) with New York Heart Association class II, III, or IV HF and an ejection fraction of 40% or less to receive dapagliflozin 10mg once daily or placebo. Compared with placebo users, the HR for the composite of CV outcomes, worsening heart failure, cardiovascular death, and die from any causes of dapagliflozin users were 0.74 (95%CI, 0.65-0.85), 0.70 (95% CI: 0.59 to 0.83), 0.82 (95% CI: 0.69 to 0.98), and 0.83 (95% CI: 0.71 to 0.97), respectively. The effects of dapagliflozin appeared consistent in patients with or without diabetes.

**SOLOIST-WHF (2020)** randomly assigned 1222 type 2 diabetes patients with recent worsening heart failure (100.0% diabetic, 69.9% CKD) to receive sotagliflozin or placebo. Compared with placebo sotagliflozin users had significantly lower risk of the composite of CV outcomes (HR: 0.67, 95% CI: 0.52 to 0.85) but had similar risk of cardiovascular death (HR: 0.84, 95% CI: 0.58 to 1.22) and death from any causes (HR: 0.82, 95% CI: 0.59 to 1.14). Moreover, sotagliflozin users also had greater risk of diarrhea, severe hypoglycemia than placebo users.

**EMPEROR-Reduced Trial** **(2020)** randomized 3,730 HF patients (49.8% diabetic, 48.3% CKD) with an ejection fraction of 40% or less into two groups that received empagliflozin 10mg once daily and placebo separately. Compared with placebo users, empagliflozin users had significantly lower risk for the composite of CV outcomes (HR: 0.75, 95% CI: 0.65 to 0.86). The annual rate of eGFR decline was significnalty slower in the empagliflozin group than in the placebo group (–0.55 vs. –2.28 ml/min/1.73m^2^, P<0.001). In the subgroup analysis, non-diabetic individuals with empagliflozin had a reduced risk of 22% than placebo for the primary composite outcomes. (HR: 0.78, 95% CI: 0.64–0.97).

**EMPEROR-Preserved Trial** **(2021)** randomly assigned 5,988 HF patients (49.1% diabetic, 49.9% CKD) with an ejection fraction of more than 40% to receive empagliflozin 10mg once daily and placebo separately. Compared with placebo users, empagliflozin users had significantly lower risk for the composite of CV outcomes (HR: 0.79, 95% CI: 0.69 to 0.90). The effects of empagliflozin appeared consistent in patients with or without diabetes.

**DELIVER trial (2022)** randomly assigned 6,263 HF patients (44.8% diabetic, 49.0% CKD) with an ejection fraction of more than 40% with dapagliflozin 10mg QD or placebo. Compared with placebo users, the HR for the composite of CV outcomes, worsening heart failure, and cardiovascular death of dapagliflozin users were 0.82 (95% CI: 0.73 to 0.92), 0.79 (95% CI: 0.69 to 0.91), and 0.88 (95% CI: 0.74 to 1.05), respectively. The effects of dapagliflozin appeared consistent in patients with a left ventricular ejection fraction of 60% or more and those with a left ventricular ejection fraction of less than 60%. Results were also similar in patients with or without diabetes.

**EMPULSE** **(2022)** randomly assigned 530 HF patients (45.3% diabetic, 9.6% advanced CKD) to receive empagliflozin (10 mg once daily) or placebo. Compared with placebo users, the stratified win ratio of the clinical benefit in composite of cardiovascular outcomes of empagliflozin users was 1.36 (95% CI: 1.09 to 1.68).

**For analysis of the clinical effect of SGLT-2 inhibitors for patients with chronic kidney disease, 4 studies were included.**

**CREDENCE (2019)** randomly assigned 4401 type 2 diabetic nephropathy participants (100.0% diabetic, 14.8% heart failure) with an eGFR of 30 to < 90 ml/min/1.73m^2^ and a urinary albumin-to-creatinine ratio of >300 to 5000 and were treated with renin–angiotensin system blockade to receive canagliflozin (100 mg once daily) or placebo. Compared with placebo users, the HR for the composite of kidney outcomes, end-stage kidney disease and the composite of CV outcomes of canagliflozin users were 0.70 (95% CI: 0.59 to 0.82), 0.68 (95% CI: 0.54 to 0.86), and 0.80 (95% CI: 0.67 to 0.95), respectively.

**DAPA-CKD (2020)** randomly assigned 4304 CKD participants (67.5% diabetic, 37.4% heart failure) with an eGFR of 25 to 75 ml/min/1.73m^2^ and a urinary albumin-to-creatinine ratio of 200 to 5000 to receive dapagliflozin (10 mg once daily) or placebo. It showed a significantly lower risk of CKD progression or death from kidney or cardiovascular causes in a mixed population of patients with diabetic and non-diabetic CKD receiving dapagliflozin in comparison with placebo (HR: 0.61, 95% CI: 0.51 to 0.72). In the subgroup analysis, compared with placebo users, the HR for the composite of kidney outcomes, composite of CV outcomes, and death of dapagliflozin users were 0.56 (95% CI: 0.45 to 0.68), 0.71 (95% CI: 0.55 to 0.92), and 0.69 (95% CI: 0.53 to 0.88), respectively. The beneficial effects of dapagliflozin were similar in participants with and without diabetes.

**SCORED (2021)** randomly assigned 10584 type 2 diabetic nephropathy participants (100.0% diabetic, 31.0% heart failure) with an glycated hemoglobin level ≥7%, eGFR of 25 to 60 ml/min/1.73m^2^ and risk for cardiovascular disease to receive sotagliflozin or placebo. Compared with placebo sotagliflozin users had significantly lower risk of the composite of CV outcomes (HR: 0.74, 95% CI: 0.63 to 0.88) but had similar risk of cardiovascular death (HR: 0.90, 95% CI: 0.73 to 1.12) and MACE (HR: 0.84, 95% CI: 0.72 to 0.99). Moreover, sotagliflozin users also had greater risk of diarrhea, genital mycotic infections, volume depletion, and diabetic ketoacidosis than placebo users.

**EMPA-KIDNEY (2022)** randomly assigned 6609 CKD participants (46.0% diabetic) with an GFR of 20 to 45 ml/min/1.73m^2^ or an eGFR of 45 to 90 ml/min/1.73m^2^ with a urinary albumin-to-creatinine ratio of at least 200 to receive empagliflozin (10 mg once daily) or placebo. It showed a significantly lower risk of composite kidney outcomes in empagliflozin users, compared with placebo users (HR: 0.72, 95% CI: 0.64 to 0.82).

**9. PRISMA checklist**

| **Section and Topic** | **Item #** | **Checklist item** | **Location where item is reported** |
| --- | --- | --- | --- |
| **TITLE** | | |  |
| Title | 1 | Identify the report as a systematic review. | Page 1 |
| **ABSTRACT** | | |  |
| Abstract | 2 | See the PRISMA 2020 for Abstracts checklist. | Page 2 |
| **INTRODUCTION** | | |  |
| Rationale | 3 | Describe the rationale for the review in the context of existing knowledge. | Page 3-4 |
| Objectives | 4 | Provide an explicit statement of the objective(s) or question(s) the review addresses. | Page 3-4 |
| **METHODS** | | |  |
| Eligibility criteria | 5 | Specify the inclusion and exclusion criteria for the review and how studies were grouped for the syntheses. | Page 5-6 |
| Information sources | 6 | Specify all databases, registers, websites, organisations, reference lists and other sources searched or consulted to identify studies. Specify the date when each source was last searched or consulted. | Page 5-6 |
| Search strategy | 7 | Present the full search strategies for all databases, registers and websites, including any filters and limits used. | Page 5-6 |
| Selection process | 8 | Specify the methods used to decide whether a study met the inclusion criteria of the review, including how many reviewers screened each record and each report retrieved, whether they worked independently, and if applicable, details of automation tools used in the process. | Page 6 |
| Data collection process | 9 | Specify the methods used to collect data from reports, including how many reviewers collected data from each report, whether they worked independently, any processes for obtaining or confirming data from study investigators, and if applicable, details of automation tools used in the process. | Page 6 |
| Data items | 10a | List and define all outcomes for which data were sought. Specify whether all results that were compatible with each outcome domain in each study were sought (e.g. for all measures, time points, analyses), and if not, the methods used to decide which results to collect. | Page 6 |
|  | 10b | List and define all other variables for which data were sought (e.g. participant and intervention characteristics, funding sources). Describe any assumptions made about any missing or unclear information. | Page 6 |
| Study risk of bias assessment | 11 | Specify the methods used to assess risk of bias in the included studies, including details of the tool(s) used, how many reviewers assessed each study and whether they worked independently, and if applicable, details of automation tools used in the process. | Page 7 |
| Effect measures | 12 | Specify for each outcome the effect measure(s) (e.g. risk ratio, mean difference) used in the synthesis or presentation of results. | Page 7-8 |
| Synthesis methods | 13a | Describe the processes used to decide which studies were eligible for each synthesis (e.g. tabulating the study intervention characteristics and comparing against the planned groups for each synthesis (item #5)). | Page 7-8 |
|  | 13b | Describe any methods required to prepare the data for presentation or synthesis, such as handling of missing summary statistics, or data conversions. | Page 7-8 |
|  | 13c | Describe any methods used to tabulate or visually display results of individual studies and syntheses. | Page 7 |
|  | 13d | Describe any methods used to synthesize results and provide a rationale for the choice(s). If meta-analysis was performed, describe the model(s), method(s) to identify the presence and extent of statistical heterogeneity, and software package(s) used. | Page 7-8 |
|  | 13e | Describe any methods used to explore possible causes of heterogeneity among study results (e.g. subgroup analysis, meta-regression). | Page 7-8 |
|  | 13f | Describe any sensitivity analyses conducted to assess robustness of the synthesized results. | nil |
| Reporting bias assessment | 14 | Describe any methods used to assess risk of bias due to missing results in a synthesis (arising from reporting biases). | Page 6-7 |
| Certainty assessment | 15 | Describe any methods used to assess certainty (or confidence) in the body of evidence for an outcome. | Page 8 |
| **RESULTS** | | |  |
| Study selection | 16a | Describe the results of the search and selection process, from the number of records identified in the search to the number of studies included in the review, ideally using a flow diagram. | Page 8  Figure 1 |
|  | 16b | Cite studies that might appear to meet the inclusion criteria, but which were excluded, and explain why they were excluded. | Page 8 |
| Study characteristics | 17 | Cite each included study and present its characteristics. | Page 9-10  Table 1 |
| Risk of bias in studies | 18 | Present assessments of risk of bias for each included study. | Page 12 |
| Results of individual studies | 19 | For all outcomes, present, for each study: (a) summary statistics for each group (where appropriate) and (b) an effect estimate and its precision (e.g. confidence/credible interval), ideally using structured tables or plots. | Page 12-14 |
| Results of syntheses | 20a | For each synthesis, briefly summarise the characteristics and risk of bias among contributing studies. | Page 12 |
|  | 20b | Present results of all statistical syntheses conducted. If meta-analysis was done, present for each the summary estimate and its precision (e.g. confidence/credible interval) and measures of statistical heterogeneity. If comparing groups, describe the direction of the effect. | Page 12-14  Table 2 |
|  | 20c | Present results of all investigations of possible causes of heterogeneity among study results. | Page 12 |
|  | 20d | Present results of all sensitivity analyses conducted to assess the robustness of the synthesized results. | nil |
| Reporting biases | 21 | Present assessments of risk of bias due to missing results (arising from reporting biases) for each synthesis assessed. | nil |
| Certainty of evidence | 22 | Present assessments of certainty (or confidence) in the body of evidence for each outcome assessed. | Supplement 5 |
| **DISCUSSION** | | |  |
| Discussion | 23a | Provide a general interpretation of the results in the context of other evidence. | Page 20 |
|  | 23b | Discuss any limitations of the evidence included in the review. | Page 23 |
|  | 23c | Discuss any limitations of the review processes used. | Page 23 |
|  | 23d | Discuss implications of the results for practice, policy, and future research. | Page 24 |
| **OTHER INFORMATION** | | |  |
| Registration and protocol | 24a | Provide registration information for the review, including register name and registration number, or state that the review was not registered. | Supplement 6 |
|  | 24b | Indicate where the review protocol can be accessed, or state that a protocol was not prepared. | Supplement 6 |
|  | 24c | Describe and explain any amendments to information provided at registration or in the protocol. | Supplement 6 |
| Support | 25 | Describe sources of financial or non-financial support for the review, and the role of the funders or sponsors in the review. | Page 29 |
| Competing interests | 26 | Declare any competing interests of review authors. | Page 29 |
| Availability of data, code and other materials | 27 | Report which of the following are publicly available and where they can be found: template data collection forms; data extracted from included studies; data used for all analyses; analytic code; any other materials used in the review. | Page 30 |
